# Anion-selective Formate/nitrite transporters: taxonomic distribution, phylogenetic analysis and subfamily-specific conservation pattern in prokaryotes

**DOI:** 10.1186/s12864-017-3947-4

**Published:** 2017-07-24

**Authors:** Mishtu Mukherjee, Manu Vajpai, Ramasubbu Sankararamakrishnan

**Affiliations:** Department of Biological Sciences and Bioengineering, Indian Institute of Technology Kanpur, Kanpur, -208016 India

**Keywords:** Anion transport, Structure-based sequence alignment, FNT family, Channel selectivity and transport, Phylogenetic analysis, Hour-glass helical fold, Sequence diversity, Homology modeling, Gating, Conformational changes

## Abstract

**Background:**

The monovalent anions formate, nitrite and hydrosulphide are main metabolites of bacterial respiration during anaerobic mixed-acid fermentation. When accumulated in the cytoplasm, these anions become cytotoxic. Membrane proteins that selectively transport these monovalent anions across the membrane have been identified and they belong to the family of Formate/Nitrite Transporters (FNTs). Individual members that selectively transport formate, nitrite and hydrosulphide have been investigated. Experimentally determined structures of FNTs indicate that they share the same hourglass helical fold with aquaporins and aquaglyceroporins and have two constriction regions, namely, cytoplasmic slit and central constriction. Members of FNTs are found in bacteria, archaea, fungi and protists. However, no FNT homolog has been identified in mammals. With FNTs as potential drug targets for many bacterial diseases, it is important to understand the mechanism of selectivity and transport across these transporters.

**Results:**

We have systematically searched the sequence databases and identified 2206 FNT sequences from bacteria, archaea and eukaryotes. Although FNT sequences are very diverse, homology modeling followed by structure-based sequence alignment revealed that nearly one third of all the positions within the transmembrane region exhibit high conservation either as a group or at the level of individual residues across all three kingdoms. Phylogenetic analysis of prokaryotic FNT sequences revealed eight different subgroups. Formate, nitrite and hydrosulphide transporters respectively are clustered into two (FocA and FdhC), three (NirC-α, NirC-β and NirC-γ) and one (HSC) subfamilies. We have also recognized two FNT subgroups (YfdC-α and YfdC-β) with unassigned function. Analysis of taxonomic distribution indicates that each subfamily prefers specific taxonomic groups. Structure-based sequence alignment of individual subfamily members revealed that certain positions in the two constriction regions and some residues facing the interior show subfamily-specific conservation. We have also identified examples of FNTs with the two constriction regions formed by residues that are less frequently observed. We have developed dbFNT, a database of FNT models and associated details. dbFNT is freely available to scientific community.

**Conclusions:**

Taxonomic distribution and sequence conservation of FNTs exhibit subfamily-specific features. The conservation pattern in the central constriction and cytoplasmic slit in the open and closed states are distinct for YfdC and NirC subfamilies. The same is true for some residues facing the interior of the transporters. The specific residues in these positions can exert influence on the type of solutes that are transported by these proteins. With FNTs found in many disease-causing bacteria, the knowledge gained in this study can be used in the development and design of anti-bacterial drugs.

**Electronic supplementary material:**

The online version of this article (doi:10.1186/s12864-017-3947-4) contains supplementary material, which is available to authorized users.

## Background

A series of redox reactions take place during cellular respiration of both prokaryotes and eukaryotes. Carbon dioxide, oxidized sulfur and nitrogen compounds are the terminal electron acceptors used in the respiration of prokaryotes [1–3]. Under anaerobic conditions, the major metabolites produced during bacterial respiration include formate (HCOO^−^) and nitrite (NO_2_^−^) [4, 5]. In the mixed acid-fermentation of enterobacteria, formate plays a prominent role [5] and it is also used for energy generation by lyase reactions [6]. Another major metabolite is hydrogen sulfide and is important for the growth of anaerobic bacteria. Intracellularly produced hydrogen sulfide is dissociated into hydrosulfide (HS^−^) anion [2, 7]. Accumulation of anions such as formate, nitrite and hydrosulfide in cytoplasm leads to lethal acidification and hence the cells must find a way to export them outside the cytoplasm. Membrane proteins that are involved in the transport of anions are the ideal candidates to perform this task. Proteins that are involved in the transport of formate ions were first identified in the early 1990s [8, 9] and later nitrite and hydrosulphide-transporting proteins were also discovered [1, 10, 11]. Integral membrane proteins that are involved in the transport of formate, nitrite and hydrosulphide anions belong to the same family called formate/nitrate transporters (FNTs) and the transporter classification ID for the FNT family in the Transporter Classification Database is 1.A.16 [12]. FocA, NirC and HSC proteins are prototype members of formate, nitrite and hydrosulphide transporters respectively. They have been shown to selectively transport monovalent anions [13, 14]. Members of FNTs have been identified in bacteria, archaea, fungi and protists and they seem to be completely absent in higher eukaryotes. Organisms containing FNT members include pathogenic parasites and fungi such as *Toxoplasma gondii*, *Candida albicans*, *Aspergillus fumigatus* and *Aspergillus flavus*. They cause toxoplasmosis, oral or esophageal infection, candidiasis, lung infection, chronic pulmonary infection, vaginal yeast infection and allergic bronchopulmonary aspergillosis [15–20]. The genome of malaria parasite *Plasmodium* spp. also contains FNT ortholog [21, 22]. Since no FNT ortholog has been discovered in humans, FNT homologs from human pathogenic bacteria and fungi can be considered as attractive targets for development of antibacterial and antifungal drugs. If FNT members are to be considered as drug targets, then the mechanism of transport and selectivity for the monovalent anions have to be elucidated. Although both computational and experimental studies have been carried out to elucidate the transport mechanism of FNTs [13, 23, 24], it is still debatable whether FNTs can be described as trasnporters or channels. Hence in this paper, we have also used the term “channel” to describe the FNT members.

**Fig. 1:**
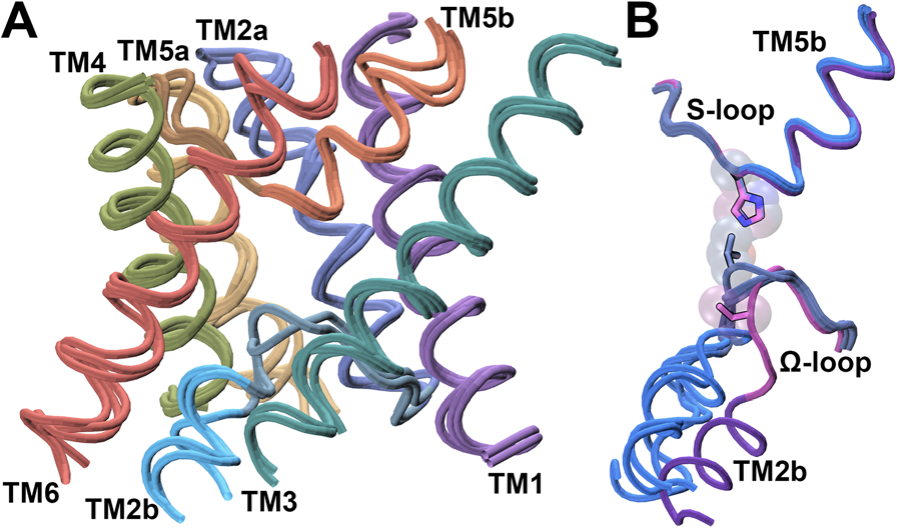
Superposition of FNT channel structures. (**a**) Superposition of experimentally determined structures of formate (PDB ID: 3KCU; monomer B;), nitrite (PDB ID: 4FC4; monomer A;) and hydrosulphide (PDB ID: 3TDR; monomer I;) channel structures. Only the backbone of six transmembrane helical segments and the Ω- and S-loops were considered for superposition. Side-chains and the loops connecting the transmembrane segments are not shown for clarity. (**b**) Superposition of functionally important Ω- and S-loops from five different monomers from three formate channel structures. Monomer A from PDB ID: 3KCU, monomers A and E from PDB ID: 3KLY and monomers A and B from PDB ID: 3Q7K were superposed in the same way as described in Fig. 1A. Only the backbone of the two loop regions is displayed to illustrate the conformational changes of Ω-loop. Molecules plotted in this figure and subsequent figures utilized the VMD software package [72]. Thr-91 from the Ω-loop and His-209 from the S-loop are shown in stick representation. It should be noted that Thr-91 has moved away representing the open state of FNT structure [26]

Knowledge of three-dimensional structures of the FNT members is key to understanding the molecular mechanism of the function of FNTs. Three-dimensional structures of selected members of FNT members have been determined. Structure of the first member of FNTs, FocA, was determined from *Escherichia coli* (PDB ID: 3KCU) [25]. Since then structures of four more homologs are available from *Vibrio cholerae* (FocA – PDB ID: 3KLY and 3KLZ) [26], *Salmonella typhimurium* (FocA – PDB ID: 3Q7K; NirC – PDB ID: 4FC4) [27, 28] and *Clostridium difficile* (HSC – PDB ID: 3TDO, 3TDR and 3TDP) [11]. Surprisingly, the structures of FNT family members exhibit the same hourglass helical fold (Fig. 1A) as that of aquaporin and aquaglyceroporin, both members of the Major Intrinsic Protein (MIP) superfamily [29–31]. The sequence identity between the two groups of channels is only less than 15%. As in MIP channel members, the characteristic hour-glass helical fold in FNT structures has six transmembrane helices (TM1 to TM6). The two transmembrane helices TM2 and TM5 are broken in the middle of the transmembrane region (TM2a and TM2b; TM5a and TM5b). TM2a and TM2b are connected by the Ω-loop and TM5a and TM5a are linked by the S-loop [26]. As in the MIP family, FNT structure exhibits a pseudo two-fold symmetry. FNT structures exhibit two constrictions called cytoplasmic slit and central constriction region. The central constriction is formed by two Phe residues from TM2a (Phe-75) and TM5a (Phe-202), one His from the S-loop (His-209) and an Ala residue from TM5b (Ala-212). The residue numbers corresponding to the PDB ID: 3KCU [25] will be used throughout the paper. The second constriction near the cytoplasmic side is formed by two Leu residues from TM2a (Leu-79) and Ω-loop (Leu-89), a Val from TM4 (Val-175) and a Thr from the Ω-loop (Thr-91). The structure of FocA from *Vibrio cholerae* determined at two different formate ion concentrations has suggested that Ω-loop can undergo conformational changes to act as a gate [26](Fig. 1b). When the gate is open, it has been suggested that the Thr-91 residue of the Ω-loop will move away and the narrow region will be formed by five residues. In addition to Leu-79 (TM2a), Leu-89 (Ω-loop) and Val-175 (TM4), a Phe residue preceding Thr-91 from Ω-loop and an Asn from TM4 (Asn-172) will form the cytoplasmic slit in the open conformation. Other residues also form part of these narrow constriction regions due to their close proximity to Phe residues at the central constriction or Leu residues at the cytoplasmic slit and this occurs at least either in the open state or in the closed state. The important residues forming the two constriction sites are mostly hydrophobic in nature and are mainly contributed by TM2 and TM5 helical segments and the two loop regions. The distance between the two constrictions is approximately 8 Ǻ. The pore diameter becomes narrow in both sites and between the two sites, cytoplasmic slit has the narrowest point within the pore (1.2 to 1.4 Ǻ).

In this paper, we have exhaustively searched sequence databases to identify FNT sequence members. We have examined the taxonomic distribution of these FNTs and analyzed the sequence conservation in the transmembrane regions of all FNT members across bacteria, archaea and eukaryotes. Conservation, either as an individual residue or as a group, is observed at specific positions of FNT channel family across the diverse organisms. We then modeled each FNT member using the known crystal structures as templates. In the case of FocA-like formate channels, structures corresponding to both open and closed states were separately used as templates to model both states. The identified FNT members from the sequence search were subjected to rigorous phylogenetic analysis. We have identified eight distinct FNT subfamilies. We used structure-based sequence alignment to identify conserved motifs specific to a particular FNT subfamily. This study helped us to discover FNT members with rare substitutions in the two constriction regions.

## Results and discussion

We searched UniProt database [32, 33] as described in the Methods section. The initial search yielded 8943 FNT sequences. After removing the redundancy at 98% level, the number of sequences has come down to 2429. We found that 95 of them have missing transmembrane segments and hence we discarded them. Another 128 sequences were also removed from the dataset because they either showed short transmembrane helices or it was not possible to align them with other FNT sequences. Thus the final dataset contained 2206 sequences, 2043 prokaryotic and 163 eukaryotic FNTs. We then used operon identification as described in the Methods section to validate as many FNT sequences as possible. We found that 15% (304 out of 2043) of the prokaryotic FNT members are associated with the known operons. Majority of 246 FNT genes are linked to either *pfl* operon (126 formate channels) or *nirBD* operon (120 nitrite channels).

## Taxonomy distribution of FNT family

**Fig. 2:**
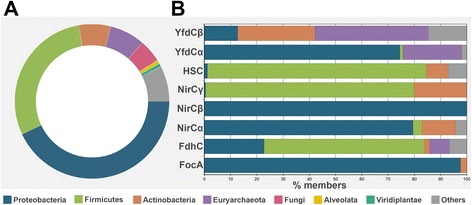
Taxonomic distribution of FNTs. (**a**) Taxonomic distribution of all 2206 FNT sequences. (**b**) Taxonomic distribution for each individual prokaryotic FNT subfamily is provided. The species groups to which the channels belong are shown in different colors

We analyzed the taxonomical distribution of FNT members. We found that about 85% of them belong to bacteria. The remaining members were equally distributed between archaea and eukarya. Taxonomical distribution of FNT channels across the three different kingdoms is shown in Fig. 2A. Half of all bacterial FNTs belong to gram-negative bacteria (proteobacteria) and about 42% of bacterial FNTs come from gram-positive bacteria (firmicutes and actinobacteria). Among the 164 archaeal FNTs, almost all of them are from euryarchaeota. The eukaryotic FNT members are majorly contributed by fungi (106 out of 163). Some FNT members are found in the single-celled alveolata and viridiplantae. Thus FNTs are primarily found in bacteria and as reported in other studies, this study confirms that no FNT members have been identified in higher animals.

## Conservation of specific residues

FNT sequences are diverse and if we simply compare all possible pairs of FNT sequences, the average sequence identity and sequence similarity are about 25% and 39% respectively. With such a huge variation in the sequences, it will be interesting to see whether any sequence positions are strictly conserved. Although the FNT sequences are diverse, they adopt a unique hour-glass helical fold shared by the MIP family [29–31]. Superimposition of experimentally determined structures of formate, nitrite and hydrosulphide channels reveal that all three adopt the same helical fold (Fig. 1A). For example, the sequence identity between the FocA formate channel from *E. coli* and nitrite channel NirC from *S. typhimurium* is about 27% in the transmembrane region and the three dimensional structures of both (PDB IDs: 3KCU; monomer B and 4FC4; monomer A) [25, 28] have the same fold with RMSD 1.6 Ǻ. If we use conventional multiple sequence alignments for all the identified FNTs, it is likely that a meaningful multiple sequence alignment cannot be produced for such diverse set of sequences. Hence, we decided to use structure-based sequence alignments as such an approach was highly successful in identifying conserved residue positions among the Major Intrinsic Protein (MIP) family members [34–36]. We modeled each member of FNT family using homology modeling technique as described in the Methods section. We then aligned each transmembrane helical region and the two functionally important loop regions (Ω and S). Residues defining the secondary structures within the transmembrane region are as per the 3KCU numbering (TM1: 31–56; TM2a: 64–85; Ω-loop: 86–93; TM2b: 94–101; TM3: 107–134; TM4: 161–184; TM5a: 188–204; S-loop: 205–209; TM5b: 210–224; TM6: 247–275). We looked at two main features, namely, group conservation of small and weakly polar residues at the helix-helix interface and the conservation of specific residues in the transmembrane region with a particular focus at the two constriction regions. This approach has previously helped to identify that several positions at the helix-helix interface are small and weakly polar in nature and as a group they show almost 90 to 100% conservation in the diverse MIP superfamily of channels that are involved in the transport of water and neutral solutes [34–36].

### Group conservation of small and weakly polar residues

Structure-based sequence alignment of all 2206 FNT sequences shows that 15 positions are highly group-conserved with small and weakly polar residues occurring at the interface of two transmembrane helical segments (Table 1). We observe more than 90% group conservation for 10 positions which are occupied by Ala, Ser, Thr, Cys or Gly. We have previously postulated that such high level of conservation of small and weakly polar residues at the interface enable the transmembrane helices to approach close together and this will help in tight packing of helices [34]. In addition to these positions, four residues from TM1, TM4 and TM6, also show high group conservation of small and weakly polar residues and they occur between TM1/TM4/TM6 and the functionally important Ω/S-loop regions (Table 1). Positions showing high group conservation of small and weakly polar residues in FNT structure are shown in Fig. S1A (see Additional file 1).

**Table 1:** Group conservation of small and weakly polar residues at the helix-helix interface of all FNT channels^a^

Residue^b^	Location in the channel	Residues in the position^c^	Conservation (%)^d^
A38 (TM1)	TM1-TM3 interface	A(46), S, G	98.2
A41 (TM1)	TM1-Ω interface	A(81), G, S	99.2
G42 (TM1)	TM1-TM3 interface	G(95), C, S	99.6
S46 (TM1)	TM1-TM3 interface	G(38), A, S, T	97.5
A48 (TM1)	TM1-TM2a interface	G(44), A(31), S, T	95.6
G72 (TM2a)	TM2a-TM5a interface	G(43), A(36), S	89.8
G78 (TM2a)	TM1-TM2a interface	G(78), A, S	99.7
G120 (TM3)	TM1-TM3 interface	G(71), A, S, C, T	87.3
G124 (TM3)	TM1-TM3 interface	G(99)	99.9
A125 (TM3)	TM1-TM5b interface	A(45), G, S, T, C	97.4
V129 (TM3)	TM1-TM5b interface	A(62), G, S, C, T	76.8
G168 (TM4)	TM4-TM6 interface	G(69), A, S	100.0
C176 (TM4)	TM4-TM6/Ω interface	C(55), A, S, G, T	89.5
A178 (TM4)	TM4-TM5a interface	A(60), S, G	79.9
G220 (S-loop)	TM1-TM5b interface	G(42), S, A, T	78.8
G261 (TM6)	TM4-TM6 interface	G(99)	99.4
G265 (TM6)	TM4-TM6 interface	G(95), S, A	99.9
G266 (TM6)	Ω-TM6 interface	G(97), A	100.0
G271 (TM6)	Ω-TM6 interface	G(56), A, T, S, C	94.7

^a^All 2206 FNT channels from bacteria, archaea and eukaryotes were considered for this analysis

^b^Residue numbers correspond to that of formate channel structure as available in the PDB ID: 3KCU. Occurrence of these residues in the transmembrane segments or the functionally important loop region is also provided

^c^Percentage conservation of the most frequently observed residue(s) is indicated in brackets

^d^Percentage conservation as observed in all 2206 FNT channels

### Conservation at the functionally important constriction regions

The three-dimensional structures of FNT members show that there are two narrow constriction regions within the channel. The cytoplasmic slit and the central constriction site are formed by mostly hydrophobic residues. The central constriction region is formed by four residues and three of them are hydrophobic (Phe-75 from TM2a, Phe-202 from TM5a, His-209 from the S-loop and Ala-212 from TM5b in 3KCU numbering). Among them, His-209 residue is almost invariant and the aromatic character of Phe-75 is conserved in nearly 99% of more than 2200 FNT sequences (Table 2). The other two residues, Phe-202 and Ala-212, also exhibit conservation more than 70%. The second constriction region, cytoplasmic slit, is formed by Leu-79 from TM2a, Leu-89 and Thr-91 from Ω-loop and Val-175 from TM4 when FNT channel is in the closed state. Among these four residues, Leu-89 and Thr-91 from the Ω-loop are nearly invariant and exhibit more than 98% conservation in all the FNT sequences analyzed in this study. Similarly, the hydrophobic character of Val-175 shows absolute conservation (Table 2). Since Ω-loop has been shown to undergo conformational changes (Fig. 1B) and is suggested to act as a gate [26], the structure determined in this condition is considered as open state. In this conformation, two other residues become part of the cytoplasmic slit (Phe-90 from Ω-loop and Asn-172 from TM4) while the residue Thr-91 moves away. Among these Asn-172 exhibits a conservation of 78%. Three residues (Leu-79 from TM2a, Phe-90 from Ω-loop Ala-212 from TM5b) which are part of either the central constriction region or the cytoplasmic slit do not show a very high level of conservation when all 2206 FNT sequences are considered for analyzing the conservation of important residues. The reasons for their poor conservation of these important residues can be explained based on the phylogenetic analysis of FNT sequences (see below).

**Table 2:** High conservation of residues facing the channel interior in all FNT channels^a^

Residue^b^	Residues in the position^c^	Conservation (%)^d^	Remarks^e^
I45 (TM1)	I(55), V, M, L	96.7	Hydrophobic
***F75 (TM2a)***	***F(79), Y***	***98.8***	***Part of the central constriction***
C82 (TM2a)	V(52), I, L	93.0	Hydrophobic
V83 (TM2a)	V(39), I(33), L, M	86.7	Hydrophobic
D88 (Ω-loop)	E(58), D	80.7	Acidic
***L89 (Ω-loop)***	***L(99)***	***99.9***	***Part of cytoplasmic slit (closed)***
***T91 (Ω-loop)***	***T(98)***	***97.9***	***Part of cytoplasmic slit (closed)***
S92 (Ω-loop)	G(50), S, A	79.1	End of Ω-loop; small and weakly polar
N121 (TM3)	N(99)	99.4	Hydrophilic, invariant
A171 (TM4)	C(62), S, A, G	99.9	Close to S-loop; small and weakly polar
***N172 (TM4)***	***N(78)***	***78.4***	***Part of cytoplasmic slit (open)***
***V175 (TM4)***	***V(81), I, M***	***99.9***	***Part of cytoplasmic slit (closed)***
V179 (TM4)	V(64), I, L, M	86.2	Hydrophobic
K191 (TM5a)	K(83), R, E	96.4	Basic
M195 (TM5a)	I(43), M, V, L	88.2	Hydrophobic
V199 (TM5a)	I(39), L, V	76.9	Hydrophobic
***F202 (TM5)***	***F(78)***	***78.5***	***Part of central constriction ring***
G206 (S-loop)	G(74), S, T	79.0	Small and weakly polar
***H209 (S-loop)***	***H(99)***	***98.7***	***Part of central constriction ring***
N213 (TM5b)	N(76)	76.5	Hydrophilic
N262 (TM6)	N(100)	100.0	Hydrophilic, invariant

^a^All 2206 FNT channels from bacteria, archaea and eukaryotes were considered for this analysis

^b^Residue numbers correspond to that of formate channel structure as available in the PDB ID: 3KCU. Occurrence of these residues in the transmembrane segsments or the functionally important loop regions is also indicated. Residues forming the two constriction regions are shown in bold and italic

^c^Percentage conservation of the most frequently observed residue(s) is indicated in brackets

^d^Percentage conservation as observed in all 2206 FNT channels

^e^Chemical nature or the location with respect to the channel interior of the conserved residue is mentioned

### Conservation of other residues facing the channel interior

Apart from the ten residues which form one of the constriction regions, we also analyzed other positions in which side-chains of residues face the interior of the channel. We found 14 residues with very high conservation of at least 75% (Table 2). Hydrophobic character of six residues (I-45 from TM1; Cys-82 and Val-83 from TM2a; Val-179 from TM4; Met-195 and Val-199 from TM5a) is highly conserved. Five residues that face the channel display the conservation of hydrophilic character. The acidic and basic character of Asp-88 from Ω-loop and Lys-191 from TM5a are highly preserved in all the FNT sequences analyzed (Table 2). High conservation of Asn facing the channel interior in three positions (Asn-121 from TM3, Asn-213 from TM5b and Asn-262 from TM6) has been recognized. Among them, Asn-121 and Asn-262 are nearly invariant showing near total conservation of 100% among diverse FNT sequences.

Apart from these residues, there are three positions within the channel in which group conservation of small and weakly polar residues are observed. These are Ser-92 from Ω-loop, Ala-171 from TM4 and Gly-206 from S-loop (Table 2). Ser-92, Ala-171 and Gly-206 face the channel interior. Residues facing the channel interior that exhibit a very high level of conservation in the FNT structure are displayed in Fig. S1B (see Additional file 1).

In addition to the above positions, we have identified an additional 21 positions and most of which are either at the monomer-monomer interface or lipid-exposed. We found that hydrophobic nature of these positions is at least 75% conserved in all the FNT sequences (see Additional file 2). Thus in total, 61 positions in the transmembrane region of the FNT channels show very high level of conservation. This conservation is due to a residue being invariant or conservation of hydrophobic or hydrophilic character or group-conservation of small and weakly polar residues.

## Phylogenetic analysis of FNT members

**Fig. 3:**
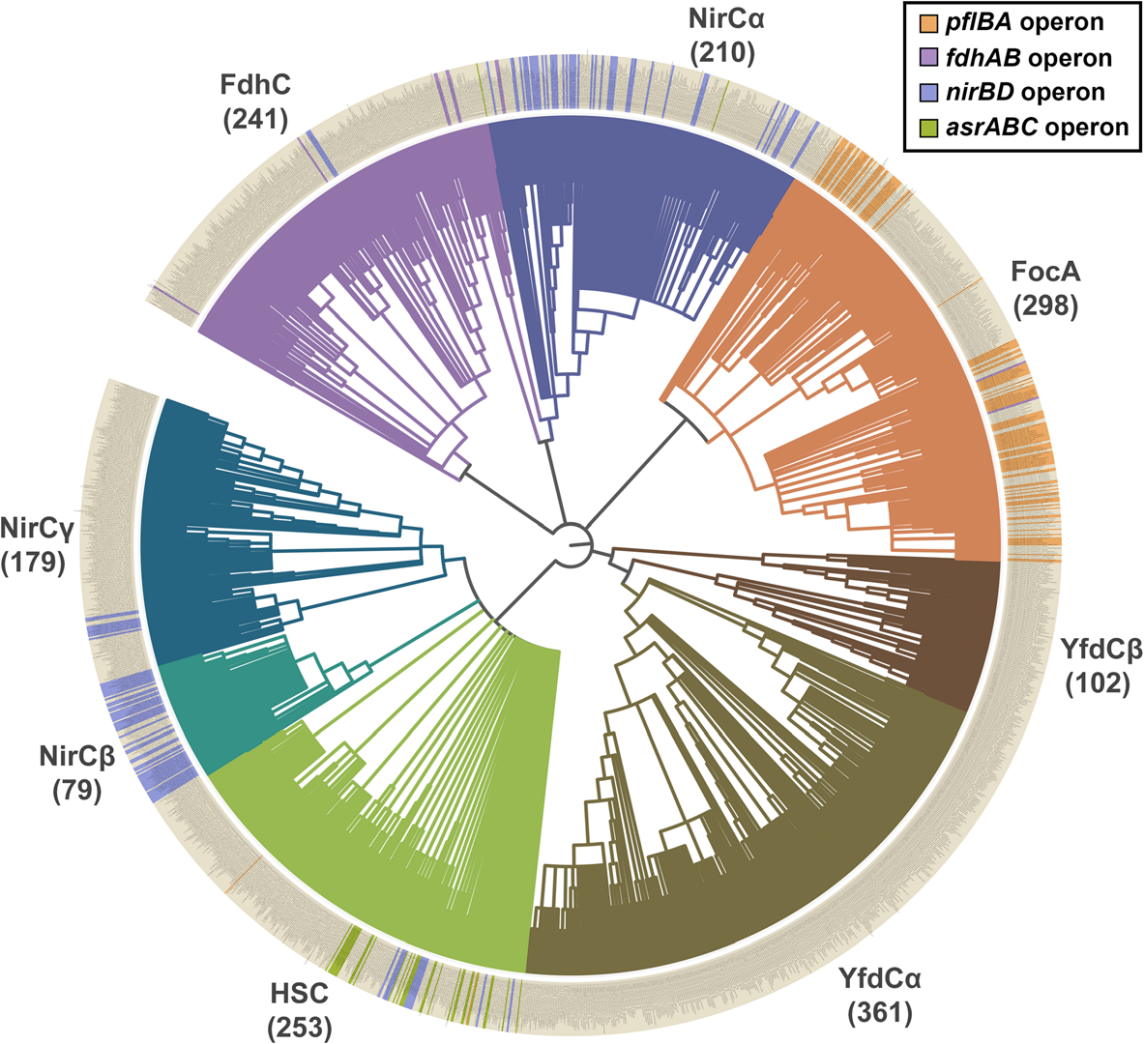
Phylogenetic analysis of prokaryotic FNTs. Phylogenetic analysis of 1723 prokaryotic FNTs identified eight subgroups. Two subfamilies (FocA and FdhC) for formate channels and three subgroups (NirC-α, NirC-β and NirC-γ) for nitrite channels have been recognized in addition to hydrosulphide (HSC) channels. The uncharacterized YfdC is divided into two clusters (YfdC-α and YfdC-β). The numbers given in the brackets represent the number of FNT channels for each cluster. With 361 and 79 members, Yfdc-α and NirC- β are the largest and smallest clusters respectively. The presence of operon is indicated in the outer circle with the respective color codes. The figure was generated using the web-based tool iTOL (Interactive Tree of Life) [73]

Analysis of more than 2200 FNT sequences identified 61 positions that displayed conservation across bacteria, archaea and eukaryotes. Czyzewski and Wang previously carried out phylogenetic studies on a limited set of 474 bacterial and archaeal FNT sequences [11] and identified five clusters including two groups of formate channels, nitrite and hydrosulphide channels. However, a large group of FNT channels remained uncharacterized with no assigned function. In this study, we have performed extensive phylogenetic analysis using two different methods on more than 2000 FNT sequences from bacteria and archaea. With large variation in loop regions, the transmembrane regions of 2043 FNT sequences from prokaryotes were used in phylogenetic studies. The tree generated by Maximum Likelihood method using RAxML [37] was validated by Bayesian inference using the program MrBayes [38] (see Methods section). Among the sequences analyzed, 320 sequences were poorly resolved with bootstrap values less than 50. Hence, we have removed those sequences from the phylogenetic analysis. The final tree produced for 1723 prokaryotic FNT sequences is shown in Fig. 3.

The phylogenetic classification of FNT sequences shows that FNT sequences are clustered into eight different groups. This includes two subfamilies of formate channels (FocA-like and FdhC-like), three clusters of nitrite channels (designated as NirC-α, NirC-β and NirC-γ), one group consisting of channels related to hydrosulphide (HSC-like) and two YfdC clusters with unknown specificity (YfdC-α and YfdC-β) [39–41]. The sequences corresponding to the FocA (PDB IDs: 3KCU, 3KLY and 3Q7K), NirC (PDB ID: 4FC4) and HSC (PDB ID: 3TDO) structures fall within the clusters of FocA, NirC-β and HSC respectively. The above grouping was further confirmed and supported by three types of analyses, namely, operon-based genetic linkage to specific enzymes, taxonomic distribution and identification of sequence motifs specific to a subfamily.

As shown in Fig. 3, several members of FocA-like sequences are supported by the genetic linkage with the metabolic enzyme pyruvate formate lyase. Similarly, most members of the nitrite channel subfamily NirC-β and many members of NirC-α have the operon support, connected by nitrite reductase. Although taxonomic distribution of NirC-γ is similar to HSC channels, it differs in residue conservation pattern in some positions (see below). Few members of NirC-γ also have operon support. Among the NirC clusters, NirC-β has the strongest support to be classified as nitrite channels. With only few members having operon support, NirC-γ is weakly supported to be grouped as one of the nitrite channel clusters. Some members of HSC-like hydrosulphide channels are linked by *asrABC* operon. Few members of the other formate channel subfamily, FdhC-like, are found to have genetic link with formate dehydrogenase. We have not found any genetic link with specific metabolic enzymes for the YfdC members. Some isolated examples of HSC-like and FdhC-like channel members are linked to metabolic enzymes such as nitrite reductase pointing to the fact that they are like nitrite channels. But their taxonomic distribution (see below) and sequence motifs indicate they display features of HSC-like or FdhC-like channels. These individual members should be experimentally characterized to find out the reasons for their genetic link with different metabolic enzymes.

## Intra- and inter-group sequence comparisons of FNT channel subfamilies

One way to determine the extent of FNT family diversity is to find out the percentage sequence identity using pairwise sequence alignment. To find out the extent of diversity in each FNT subfamily, we calculated the sequence identity and similarity for every pair of FNT sequences within a subgroup and calculated the average pairwise sequence identity and similarity. Similarly we also compared two different FNT subgroups and find out the average sequence identity and similarity by considering all possible pairs between the subgroups under consideration. Only transmembrane helical regions and the loops Ω and S were considered for this purpose. Average pairwise sequence identity and similarity were calculated between each pair of all eight FNT subgroups. This data for intra- and inter- FNT subgroups are provided in Table 3. Among all FNT subfamilies, the average pairwise sequence identity for FNT sequences within NirC-β subgroups is the highest. With 85% average pairwise sequence identity and 92% sequence similarity, the sequences within this subfamily are very closely related to each other. The FNT sequences within the YfdC-β are diverse and are the most distantly related. The average pairwise sequence identity for FNT sequences within this group is only 40%. This is followed by NirC-γ and HSC subfamilies which exhibit an average pairwise sequence identity of 45%. Intra-subgroup average pairwise sequence identities for other FNT subgroups vary between 48 to 64%. When inter-subgroup pairwise sequence identities are compared, they generally vary between 25 to 35%. Only members of NirC-β share higher average pairwise sequence identity of 42% with HSC subfamily members. Thus it is clear that FNT channel members within a subgroup are generally closely related. However, when members of two FNT subfamilies are compared, they are distantly related showing that FNT family from bacteria and archaea are diverse.

**Table 3:** Average pairwise sequence identity and similarity of intra- and inter-subgroup of FNT channels shown in percentages^a,b^

	FocA	FdhC	NirC-α	NirC-β	NirC-γ	HSC	YfdC-α	YfdC-β
FocA	**64 (80)**	35 (53)	33 (53)	33 (52)	28 (46)	34 (53)	25 (44)	25 (43)
FdhC		**55 (71)**	35 (54)	33 (51)	28 (45)	32 (50)	25 (42)	26 (42)
NirC-α			**63 (78)**	29 (47)	26 (43)	28 (48)	25 (42)	23 (44)
NirC-β				**85 (92)**	29 (48)	42 (62)	24 (41)	25 (42)
NirC-γ					**45 (64)**	29 (48)	21 (38)	22 (38)
HSC						**45 (64)**	23 (42)	25 (43)
YfdC-α							**48 (66)**	30 (50)
YfdC-β								**40 (58)**

^a^Only the transmembrane helical segments and the two loop regions (Ω and S) were considered for pairwise sequence alignments

^b^Numbers shown in bold are average of percent sequence identity and similarity for all possible pairs within the specified subgroup. Sequence similarity values are shown in brackets

## Taxonomic distribution of FNT channel subfamilies

Although FNT family as a whole is almost equally divided between gram-negative and gram-positive bacteria, taxonomic distribution of FNT subfamily members show that certain FNT subfamilies are predominantly found only in specific taxonomic groups (Fig. 2B). Among the two subfamilies of formate channels, more than 95% of FocA members are found in proteobacteria. The other subgroup of formate channels, FdhC, shows a totally different taxonomic distribution. Only slightly more than 20% of FdhC members are found in gram-negative bacteria while more than 60% of them are distributed in gram-positive bacteria and they are mostly in firmicutes. A number of FdhC members are found in euryarchaeota. Among the three subfamilies of nitrite channels, NirC-β is found only in proteobacteria while NirC-γ is almost always present in gram-positive bacteria. While nearly 80% of NirC-α members are found in gram-negative bacteria, about 15% of are identified in gram-positive bacteria. Majority of members belonging to the HSC subfamily are observed in gram-positive bacteria with more than 80% of them identified in firmicutes. Most of the archaeal examples are found in the uncharacterized YfdC subfamilies. While nearly 75% of YfdC-α members are found in proteobacteria, only little more than 10% of YfdC-β members are present in gram-negative bacteria. Almost 30% of all YfdC-β members are from gram-positive bacteria. This analysis clearly shows that most of the FNT subfamilies have preference to occur either in gram-positive or gram-negative bacteria and within them they also show preference to be found in specific species groups. It also holds good for archaeal FNT members.

## Subfamily-specific sequence conservation in FNT members

Structure-based sequence alignments of individual transmembrane segments of all FNT sequences show that certain residues are invariant or exhibit near absolute conservation across different taxonomic groups that include bacteria, archaea and eukaryotes (Tables 1 and 2; see above). It is also true for the group conservation of small and weakly polar residues at the helix-helix interface. In this analysis, we performed structure-based sequence alignment for individual subfamilies of FNTs and analyzed the conserved positions. This includes the residues that form the two constriction regions, residues from the two functionally important loop regions, other residues that face the channel interior and those that preserve the conservation of small and weakly polar residues as a group. Each group of these residues is discussed in the following sections and the results of this analysis are summarized in Table 4.

**Table 4:** Subfamily-specific conservation in FNT channels^a^

Residue^b^	FocA	FdhC	NirC-α	NirC-β	NirC-γ	HSC	YfdC-α	YfdC-β
F202^c^ (TM5a)	F (100)	F (99.6)	F (100)	F (100)	F (100)	F (100)	V, I, M (99.7)	I, M, L, V (100)
A212^c^ (TM5b)	A (100)	A (93.4)	V (100)	A (100)	A (82.7)	A (100)	V (71.5)	V, I, L (73.5)
L79^d^ (TM2a)	L, V, I (99)	L, I (93.4)	F, Y (98.1)	L (100)	L (99.4)	L, V, I (96.0)	F, Y (100)	F (73.5)
F90^e^ (Ω-loop)	F (100)	V, L, I (86.3)	V, L (90.9)	F, Y (100)	---	F (91.7)	F, Y (99.7)	F (100)
N172^e^ (TM4)	N (100)	N (99.6)	N (100)	N (100)	N (99.4)	N (96.4)	G (99.7)	G (99.0)
G86 (Ω-loop)	G (99.3)	G (100)	G (91.9)	G (100)	N, Q (76)	G, S (95.6)	R, H, D (81.2)	R, H, K (75.5)
S92 (Ω-loop)	S (100)	G, S (100)	G (99.0)	G (100)	S, G (100)	G, S, A (100)	E (100)	E (90.2)
T93 (Ω-loop)	T, S (100)	N (91.3)	V (84.8)	H (98.7)	N (95.5)	N (88.9)	N (93.1)	---
K100 (TM2b)	K, R (79.9)	A, S, G (99.6)	A, S, G (99.5)	G, A (100)	G, A, S (100)	G, S, A, T (97.2)	P (75.1)	---
E208 (S-loop)	E (100)	Q (83)	E (100)	E (100)	E (99.4)	E, D (96.8)	A, S, T (75.6)	---
T34 (TM1)	T, A, S (94.3)	L, M, I, V (78.8)	---	F (100)	Y, F (98.9)	F, Y (98.8)	L, I (93.9)	L, I, V, M (94.1)
L37 (TM1)	L, M (81.2)	L (83.0)	R, K (95.2)	G, S, A (96.2)	R, K (98.9)	---	S, A (96.4)	T, S (97.1)
N113 (TM3)	N (77.2)	N (90.9)	N (74.3)	V, I (96.2)	V, L, I, M (99.4)	---	L, V, I, M (85.6)	L, I, V, (99.0)
L167 (TM4)	L (100)	S (75.1)	K, R (94.8)	K, R (100)	R, K, E, D (98.3)	R, K (95.6)	---	---
N254 (TM6)	N (100)	N (97.9)	N (100)	N (100)	N, Q (72.6)	N (89.3)	F, Y (96.1)	F, W (74.5)

^a^Percentage conservation residues for each subfamily is given in brackets

^b^Residue numbers correspond to that of formate channel structure as available in the PDB ID: 3KCU. Occurrence of these residues in the transmembrane segments or the functionally important loop regions is also indicated

^c^Part of the central constriction site; ^d^Part of the cytoplasmic slit in the closed state; ^e^Part of the cytoplasmic slit in the open state

### Residues forming the central constriction

**Fig. 4:**
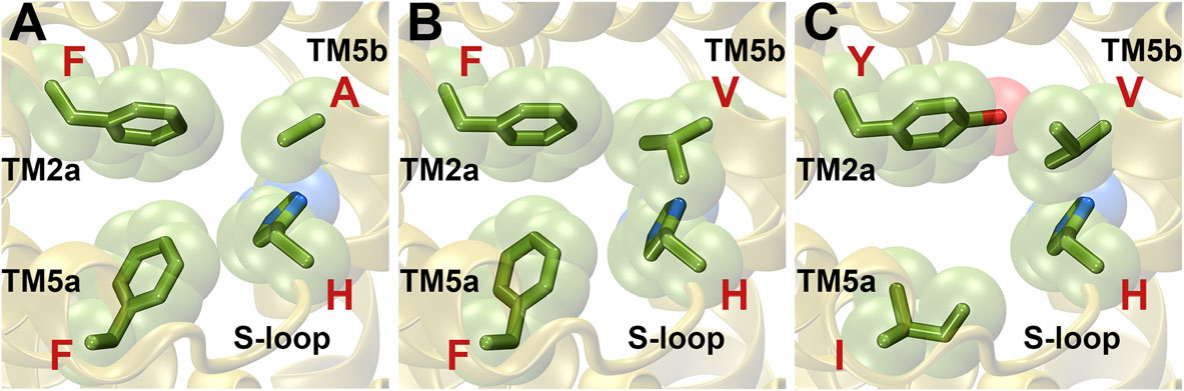
Residues showing subfamily-specific variations at the central constriction. Residues of FNT channels forming central constriction are shown for (**a**) FocA (PDB ID: 3KLY, monomer A), (**b**) NirC-α (UniProt ID: I1XLF9) and (**c**) YfdC-α (UniProt ID: U1X6D4). The four residues correspond to Phe-75 (TM2a), Phe-202 (TM5a), His-209 (S-loop) and Ala-212 (TM5b) according to the PDB structure 3KCU numbering. His-209 residue in the S-loop is almost invariant in all the FNT channels. The aromatic character of Phe-75 in TM2a shows near absolute conservation. Ala-212 in TM5b is replaced by Val in NirC-α and YfdC-α subfamilies (Table 4). Phe-202 in TM5a is substituted by other hydrophobic residues such as Ile, Val, Met and Leu in YfdC subfamilies

Four residues have been identified to form the central constriction, one of the two narrowest regions within the FNT channel. Two of the residues, Phe-75 from TM2a and His-209 from the S-loop (residue numbering is as per the PDB ID: 3KCU), have shown conservation across all the FNT subfamilies. While the aromatic character at Phe-75 exhibits more than 98% conservation, His-209 is almost invariant in all the FNT sequences (Table 2). Two other residues, Phe-202 from TM5a and Ala-212 from TM5b, display subfamily-specific conservation. While Phe at the 202 position is 100% conserved in the two formate channel subfamilies (FocA and FdhC), in the three nitrite channel subgroups (NirC-α, NirC-β and NirC-γ) and in the hydrogen sulphide channel subfamily (HSC), it is replaced by other hydrophobic residues like Val, Ile, Met and Leu in the two uncharacterized FNT subfamilies (YfdC-α and YfdC-β) (Table 4). Similarly, Ala-212 from TM5b is nearly 100% conserved in FocA, FdhC, NirC-β and HSC subfamilies. In NirC-γ, conservation of Ala is very high close to 83%. However in NirC-α, YfdC-α and YfdC-β, this Ala is substituted by Val (Table 4). While Val at this position is 100% conserved in NirC-α, it is more than 70% conserved in both the YfdC subfamilies. The replacement of Phe and Ala respectively at 202 and 212 positions in the cytoplasmic slit of the uncharacterized YfdC channels indicate that their substrate specificity is likely to be different from the other FNT channel subfamilies. Representative examples of FNT channels with subfamily-specific variations in the central constriction are shown in Fig. 4.

### Residues at the cytoplasmic slit

Among the residues that are part of the cytoplasmic slit in the closed state, Leu-89 and Thr-91 from the Ω-loop show near absolute conservation across all FNT subfamilies (Table 2). However, other residues that are part of this constriction exhibit subfamily-specific conservation indicating that these positions could be vital in determining the solute specificity that is characteristic to that subfamily. Leu-79 from TM2a is nearly 100% conserved in the two subfamilies of formate channels, NirC-β, NirC-γ and HSC subfamilies. In NirC-α and the two YfdC subfamilies, Leu is replaced by Phe and to a lesser extent by Tyr (Table 4). Phe at this position is almost 100% conserved in NirC-α and YfdC-α subfamilies. Val-175 is found to be invariant in FocA, FdhC, NirC-α and NirC-β subfamilies. In other subfamilies, the absolute conservation of hydrophobic character is found at this position indicating that hydrophobic nature of side-chain is likely to play a major role in substrate specificity (Table 2).

**Fig. 5:**
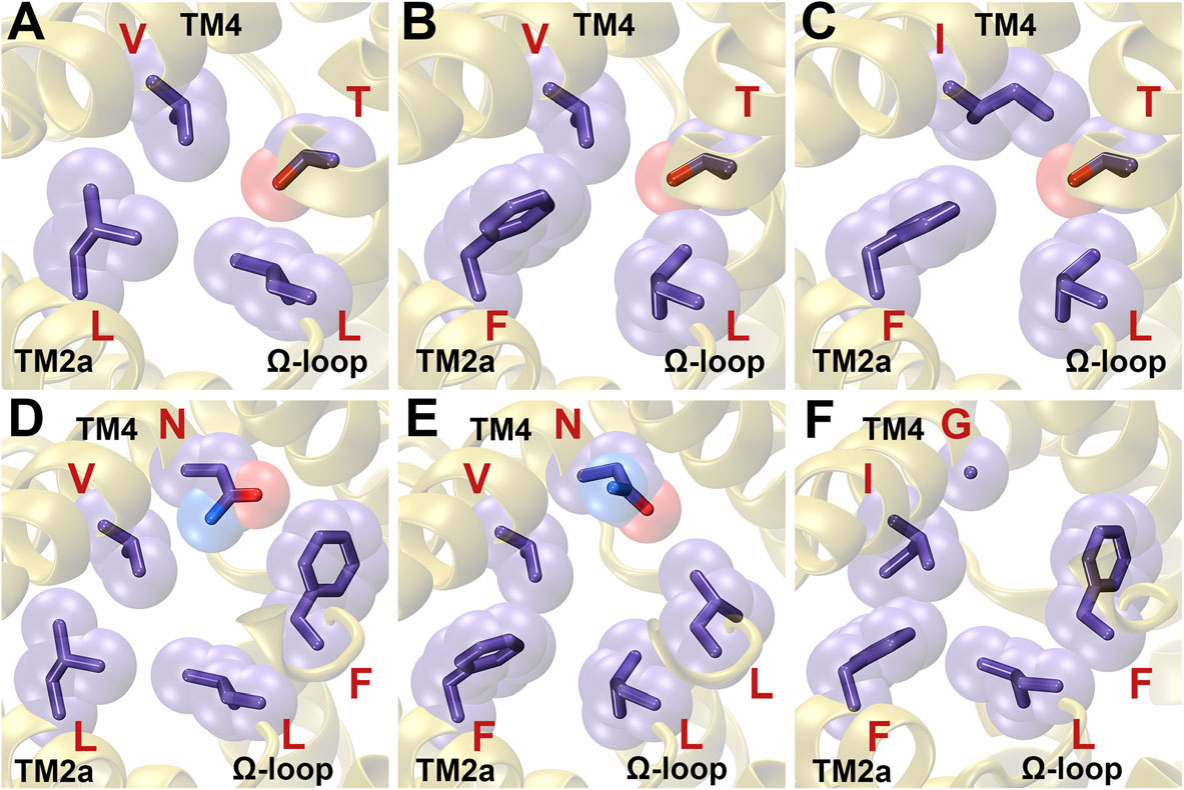
Residues displaying subfamily-specific variation at the cytoplasmic slit. Residues of FNT channels forming the cytoplasmic slit representing the (A, B, C) closed and (D, E, F) open states. The cytoplasmic slit in the closed state is shown for (**a**) FocA (PDB ID: 3KLY; monomer A), (**b**) NirC-α (UniProt ID: I1XLF9) and (**c**) YfdC-α (UniProt ID: U1X6D4). The four residues forming the cytoplasmic slit in the closed state in PDB ID: 3KCU numbering are Leu-79 (TM2a), Leu-89 (Ω-loop), Thr-91 (Ω-loop) and Val-175 (TM4). Leu-89 and Thr-91 are almost invariant across all FNT channels. The hydrophobic character of Val-175 is maintained in all FNT subfamilies. Leu-79 is substituted by an aromatic residue in NirC-α and YfdC subfamilies. The cytoplasmic slit in the open state is formed by Leu-79 (TM2a), Leu-89 (Ω-loop), Phe-90 (Ω-loop), Asn-172 (TM4) and Val-175 (TM4) and is shown for (**d**) FocA (PDB ID: 3KLY; monomer E), (**e**) NirC-α (UniProt ID: I1XLF9) and (**f**) YfdC-α (UniProt ID: U1X6D4). Open states for NirC-α and YfdC-α were modeled similar to the procedure to model the open states of FocA channel described in the Methods section. Phe-90 shows absolute conservation in FocA subfamily and the aromatic character is nearly 100% conserved in YfdC-α subfamily. However, Asn-172, while showing absolute conservation in FocA and NirC-α subfamilies, is replaced by a Gly residue in YfdC subfamilies

The functionally important Ω-loop has been shown to exist in two different conformational states and has been suggested to play a gating role [26] (Fig. 1B). In a conformational state of this loop suggested to represent the open state of the channel, Thr-91 has moved away and the residues Phe-90 from the Ω-loop and Asn-172 from TM4 have become part of the cytoplasmic slit in the open state. Phe-90 shows subfamily-specific conservation (Table 4). It is 100% conserved in FocA subfamily. A high conservation of more than 90% is also observed for Phe/Tyr in NirC-β, HSC, YfdC-α and YfdC-β subfamilies. In FdhC and NirC-α subfamilies, Phe is replaced by other hydrophobic residues such as Val, Leu and Ile. No specific residues are conserved in this position in NirC-γ subfamily. Asn-172 displays absolute or near absolute conservation in the subfamilies of formate, nitrite and hydrogen sulphide channels. However, in the uncharacterized YfdC subfamilies, this Asn is replaced by Gly with almost 100% conservation. Representative examples of FNT members displaying subfamily-specific conservation at the cytoplasmic slit at closed and open states are displayed in Fig. 5.

### Residues from S-loop and Ω-loop

Both S-loop and Ω-loop are functionally important and the residues from these loops are part of the two constriction regions. Most of the subfamily-specific conservations are observed in the Ω-loop (Table 4). The residue Gly-86 is at the very beginning of the Ω-loop near the cytoplasmic entrance of the channel. This residue is more than 90% conserved in FocA, FdhC, NirC-α, NirC-β and HSC channel subfamilies. However in NirC-γ, this position is occupied by Asn for most of the time. In YfdC families, a charged residue (mostly basic) is found indicating that this residue could influence the selectivity of the FNT channels.

The side-chain of Ser-92 faces the interior of the channel in the Ω-loop. While this position is either Ser or Gly and a near absolute conservation of these residues are found in this position, the YfdC subfamilies have Glu at this position and is found to be at least 90% conserved in these uncharacterized FNT channels (Table 4). Thr-93 is another residue which is inside the channel and is likely to influence the solute that is transported across the channel. This position is occupied by Ser/Thr in 100% of all the FocA members. However in FdhC, NirC-γ, HSC and YfdC-α subfamilies, Asn is found in at least 90% of the channels. In NirC-β, His is the preferred residue and is conserved close to 100%. In NirC-α, the hydrophobic Val is found close to 85% of the time. No specific conservation was found in YfdC-β subfamily. The location of both Ser-92 and Thr-93 residues in the Ω-loop clearly indicates that these positions are likely to have a major role in the transport and selectivity.

Lys-100 in TM2b is close to 80% conserved in FocA channels. In FdhC, in all three nitrite channel subfamilies and in HSC channels, this position is nearly 100% group conserved with small and weakly polar residues (Ala, Ser, Gly and Thr). Only in YfdC-α subfamily, Pro is found to be conserved 75% of the time. Since Lys-100 is pointing towards the channel at the cytoplasmic entrance, nature of amino acid at this position is important and that could be determining factor in the transport and selectivity of FNT channels.

One residue from the S-loop that exhibits subfamily-specific conservation is Glu-208. In FocA, in all three subfamilies of nitrite channels and in HSC channels, Glu is nearly invariant. In FdhC members, Glu is replaced by Gln and the conservation is more than 80% (Table 4). As in the previous cases, the two YfdC subgroups show marked differences. YfdC-α has mostly Thr or other small residues at this position while YfdC-β does not display preference for any specific residues.

### Other residues from the transmembrane segments facing the channel interior

We have found that few hydrophilic residues face the channel interior and show subfamily-specific conservation. The residues Thr-34 from TM1 and Asn-113 from TM3 are in close proximity with each other and they are near the cytoplasmic entrance of the channel. In FocA, Thr-34 position shows nearly 90% conservation of Thr/Ser residues and more than 75% conservation of Asn is observed at 113 position (Table 4). There is a different pattern observed for other FNT subfamilies. With the exception of NirC-α, all other subfamilies exhibit very high conservation of hydrophobic or aromatic residues at 34 position. As in FocA, Asn conservation is very high in FdhC and NirC-α at 113 position while the hydrophobic character in all the remaining subfamilies show extremely high level of conservation at the same position.

Leu-37 is the residue from TM1 near the cytoplasmic entrance facing the channel interior. While the hydrophobic character at this position is highly conserved in the two subfamilies of formate channels, this position is basic (Arg or Lys) and is more than 95% conserved in NirC-α and NirC-γ. High conservation of small residues is found for NirC-α, YfdC-α and YfdC-β subfamilies. No conservation is found for this position for the HSC subfamily indicating that this position may not play a significant role in the transport properties of HSC channels. However, the presence of basic or hydrophobic residues in some of the FNT subgroups indicates that this position can influence the selectivity of solutes in the transport of at least some of the FNT subgroups.

The TM4 helical segment also has residues that show subfamily specific conservation. The residue Leu-167 is near the periplasmic entrance of the channel and is close to the S-loop. Leu at this position is 100% conserved in FocA subfamily. However, a high conservation of Ser is found in the other formate channel subfamily FdhC. In all three nitrite channel subgroups and HSC channels, this position is occupied by a charged residue (Table 4). While basic nature of residues at this position is more than 95% conserved in NirC-α, NirC-β and HSC channels, a high conservation of Glu is observed in NirC-γ subfamily. Being close to the functionally important S-loop, the charged residues at this position is likely to play a key role. No conservation is found for the uncharacterized YfdC subfamilies.

The residue Asn-172 is facing away from the channel and is in close proximity to Asn-262 from TM6. While Asn-262 is 100% conserved in all FNT subfamilies, Asn-172 also shows the same trend for all FNT subgroups with the exception of YfdC channels. The residue Gly is almost 100% conserved in both YfdC-α and YfdC-β channels.

Asn-254 from TM6 is facing the channel at the periplasmic entrance and shows near absolute conservation in FocA, FdhC, NirC-α and NirC-β subfamilies and is highly conserved in NirC-γ and HSC channels. However, this residue is substituted by an aromatic residue in YfdC subfamilies indicating that this position could play an important role in the transport of solutes.

### Subfamily-specific features in loops and terminal regions

We have examined whether the FNT subfamilies display any characteristic features in terms of length and charge distribution in the N- and C-termini segments and in the loop regions connecting the transmembrane segments (see Table S2 in Additional file 3). This analysis does not include the functionally important Ω- and S-loops. The average length of N-terminal segment of uncharacterized YfdC subfamilies is the longest, between 42 to 55 residues, while the formate, nitrite and HSC channel subgroups possess on an average 25 to 32 amino acids in the N-terminal region before the beginning of the first transmembrane helical segment. Loop A connecting TM1 and TM2a helices is the shortest for the NirC-α subgroup. However, the same subgroup has longest Loop C linking TM3 and TM4 helices (see Table S2 of Additional file 3). The formate channel subfamily, FocA, has uncharacteristically long Loop E between TM5b and TM6 segments. The two subfamilies, FocA and YfdC-α, possess longest C-terminal segments among all prokaryotic FNT channels. We do not see any specific differences in the charge distribution between different subgroups of FNT channels. Similarly, no specific residue is conserved within a subfamily or across the different FNT subgroups in the N- or C-terminal regions.

## FNT members with unusual constriction regions

Although the residues forming the cytoplasmic slit and central constriction region are mostly invariant across all FNTs, there are some subfamily-specific variations (see above) indicating that these positions have potentially significant role in the selectivity and transport of solutes across these transporters. In our previous studies on MIP channels, we identified examples of MIP channel members with unusual selectivity filters [31]. We have applied the same strategy to identify FNTs with constriction site residues that are unique and rarely found. Two parameters, cumulative hydropathy index (CHI) and cumulative vdw volume (CVV), were used for this purpose [31]. The parameter CHI is the sum of the hydropathy values of individual residues that constitute the constriction site under consideration. It gives an idea how hydrophobic/hydrophilic is that particular constriction site. The second parameter CVV is calculated by summing the vdw radii of individual amino acid residues of the constriction site. CVV tells how occluded the constriction site is. The hydropathy scale proposed by Kyte and Doolittle [42] and the vdw volumes of amino acids as reported in Crieghton [43] were used to determine CHI and CVV values respectively.

### FNTs with unique cytoplasmic slit

**Fig. 6:**
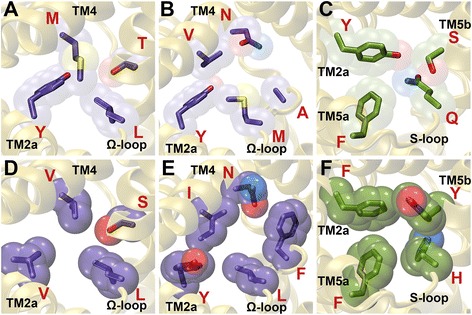
FNT channels with unique constriction sites. FNT channels with less hydrophobic cytoplasmic slit in the (**a**) closed (UniProt ID: L8XVV6 from YfdC-α; CHI value: +3.7) and (**b**) open (UniProt ID: D5BVC1 from NirC-α; CHI value: +3.1) states. (**c**) FNT channel with less hydrophobic central constriction site (UniProt ID: Q1Q4Z9- unclassified; CHI value: -2.8). (**d**) FNT channel with a wider cytoplasmic slit in the closed state (UniProt ID: M0BPP6 from YfdC-β; CVV value: 407 Ǻ^3^). (**e**) FNT channel with a narrower cytoplasmic slit in the open state (UniProt ID: I0JHL8 from an unclassified FNT channel; CVV value: 620 Ǻ^3^). (**f**) FNT channel with a narrower central constriction (UniProt ID: B9DL87 from NirC-γ; CVV value: 529 Ǻ^3^)

As mentioned earlier, the cytoplasmic slit in the closed state is formed by four residues: Leu-79 (TM2a), Leu-89 (Ω-loop), Thr-91 (Ω-loop) and Val-175 (TM4). Henceforth, these four residues are simply mentioned as LL**T**V in the order they occur in the primary sequence to represent the cytoplasmic slit in the closed state. The residue shown in bold moves away when the channel undergoes transition from the closed to the open state [26]. The typical cytoplasmic slit in all eight subgroups of FNT members in the closed state involve LL**T**V (FocA, FdhC, NirC-β, NirC-γ and HSC), FL**T**V (NirC-α), FL**T**I (YfdC-α) or FL**T**M (YfdC-β) and the CHI value varies from +7.8 to +11.1. This indicates that the cytoplasmic slit is mostly hydrophobic. Hypothetically, the most hydrophobic slit would consist of all Ile residues with CHI value +18.0. Since residues forming the cytoplasmic slit in both open and closed states in majority of FNT sequences are conserved (Tables 2 and 4), the distribution of CHI values for the closed state has a pronounced peak around +11 (data not shown). While most of the FNT channels in the closed state tend to have highly hydrophobic cytoplasmic slit, we have found examples in prokaryotic FNT members which have relatively less hydrophobic cytoplasmic slit in the closed state. In these channels, the CHI values of residues forming the cytoplasmic slit vary from +3.7 to +4.1 (Table 5). The least hydrophobic cytoplasmic slit in the closed state belong to the uncharacterized YfdC-α subfamily with CHI value +3.7 (Fig. 6A). Five examples from NirC-α subgroups have the second relatively less hydrophobic slit in the closed state. In these cases, the Phe residue which is part of the cytoplasmic slit in the closed state (FL**T**V or FL**T**I) is substituted by Tyr residue.

**Table 5:** FNT channels with unique cytoplasmic slit in the closed state

FNT subfamily	UniProt ID^a^ (Organism)	Cytoplasmic slit residues^b^	CHI/CVV value^c^
YfdC-α	I9C2K5 (*Novosphingobium sp.*) J8 W598 (*Sphingomonas sp.*) K9CVI9 (*Sphingobium yanoikuyae*) L8XVV6 (*Wohlfahrtiimonas chitiniclastica*)	YL**T**M	CHI: +3.7
NirC-α	A3WW31 (*Nitrobacter sp.*) A4BNG3 (*Nitrococcus mobilis*) D5BVC1 (*Nitrosococcus halophilus*) I4EJX4 (*Nitrolancea hollandica*) Q82WJ4 (*Nitrosomonas europaea*)	YM**T**V	CHI: +4.1
YfdC-β	A5PEF7 (*Erythrobacter sp.*) M0BPP6 (*Halovivax asiaticus*)	VL**S**V	CVV: 407 Ǻ^3^

^a^The unique UniProt Accession ID is given

^b^The four residues that form cytoplasmic slit in the closed state of the channel. They correspond to L79 from TM2a, L89 and T91 from Ω-loop and V175 from TM4 of the formate channel (PDB ID: 3KCU). The residue shown in bold moves away from the cytoplasmic slit when the Ω-loop undergoes conformational changes so that FNT channel can make a transition from the closed to open state

^c^Calculation of Cumulative Hydropathy Index (CHI) and Cumulative vdw Volume (CVV) are described in the text

In the case of open state, cytoplasmic slit is formed by five residues: Leu-79 (TM2a), Leu-89 (Ω-loop), Phe-90 (Ω-loop), Asn-172 (TM4) and Val-175 (TM4). As in the closed state, the residues forming the cytoplasmic slit will be simply referred as LL**FN**V and the residues shown in bold indicate that they are part of the cytoplasmic slit only in the open state. A typical cytoplasmic slit in the open state is formed by LL**FN**V (FocA, NirC-β and HSC), LL**IN**V (FdhC), FL**LN**V (NirC-α), LL**LN**V/LL**GN**V (NirC-γ), FL**FG**I (YfdC-α) or FL**FG**M (YfdC-β). The CHI values for these typical channels belonging to different FNT subgroups vary from +7.9 to +13.5. The distribution of CHI values for the open state shows a peak around +11 (data not shown). Examples of FNT channels which have less hydrophobic cytoplasmic slit in the open state are found. The CHI values of five channels of cytoplasmic slit in the open state are found to be +3.1 and all of them belong to NirC-α subgroup (Table 6) with YM**AN**V forming the narrow constriction (Fig. 6B). A typical NirC-α has FL**LN**V in the cytoplasmic slit at the open state. While the Asn is preserved in this region, the first and third residues are substituted by Tyr and Ala respectively thus making them relatively less hydrophobic.

**Table 6:** FNT channels with unique cytoplasmic slit in the open state

FNT subfamily	UniProt ID^a^ (Organism)	Cytoplasmic slit residues^b^	CHI/CVV value^c^
NirC-α	A3WW31(*Nitrobacter sp.*) A4BNG3 (*Nitrococcus mobilis*) D5BVC1 (*Nitrosococcus halophilus*) I4EJX4 (*Nitrolancea hollandica*) Q82WJ4 (*Nitrosomonas europaea*)	YM**AN**V	CHI: +3.1
HSC	F0JF84 (*Desulfovibrio desulfuricans*)	LL**VT**L	CHI: +14.9
NirC-γ	S4D5T6, V7ZKG7 (*Enterococcus faecalis*) S4EBH9, T2NT11 (*Enterococcus faecium*) T0U7W5 (*Enterococcus sp.*) T4JYM3 (*Peptoclostridium difficile*) U5SBH7 (*Carnobacterium inhibens*) V5XL10 (*Enterococcus mundtii*) and 41 more	LL**GN**V	CVV: 497 Ǻ^3^
Unclassified	I0JHL8 (*Halobacillus halophilus*) K2GJH2 (*Salimicrobium jeotgali*) L5N8D2 (*Halobacillus sp.*) Q8ES73 (*Oceanobacillus iheyensis*)	YL**FN**I	CVV: 620 Ǻ^3^

^a^The unique UniProt Accession ID is given

^b^The five residues that form cytoplasmic slit in the open state of the channel. They correspond to L79 from TM2a, L89 and F90 from Ω-loop and N172 and V175 from TM4 of the formate channel (PDB ID: 3KCU). Residues shown in bold will become part of the cytoplasmic slit in the open state when the Ω-loop undergoes conformational changes

^c^Calculation of Cumulative Hydropathy Index (CHI) and Cumulative vdw Volume (CVV) are described in the text

We also evaluated the CVV values of cytoplasmic slit in both open and closed states for each of the FNT channel. The distributions indicated that there are some FNT channels with wider or narrower cytoplasmic slit. The CVV values for a typical FNT channel from one of the eight subfamilies vary from 457 to 476 Ǻ^3^ for the closed state and 497 to 584 Ǻ^3^ for the open state. Distributions of CVV values for cytoplasmic slits in both open and closed states for all the FNT channels also reflect this feature. Average CVV values for closed and open states are 452.8 (± 11.8) Ǻ^3^ and 571.7 (± 20.5) Ǻ^3^ respectively. However, we have found some exceptions in which CVV values deviate from this distribution. For example, two FNT channels from the uncharacterized YfdC-β subfamily have cytoplasmic slit in the closed state formed by the residues VL**S**V with CVV value 407 Ǻ^3^ (Table 5 and Fig. 6D). The smaller CVV means the channel is wider and hence could transport bulkier solutes. Similarly in the open state, 49 FNT channels from NirC-γ have LL**GN**V in the cytoplasmic slit with CVV 497 Ǻ^3^ indicating that these channels are likely to be involved in the transport of bulkier solutes (Table 6). We have also found examples of narrower cytoplasmic slit in the open state formed by YL**FN**I from the phylogenetically unclassified FNT channels with the maximum CVV value of 620 Ǻ^3^ (Fig. 6E). These channels with narrower cytoplasmic constriction in the open state could restrict the passage of larger solutes through their channels.

### FNT channels with unique central constriction region

We have carried out a similar analysis for the central constriction region. This constriction is formed by Phe-75 (TM2a), Phe-202 (TM5a), His-209 (S-loop) and Ala-212 (TM5b). As in the case of cytoplasmic slit, the residues forming the central constriction are mentioned as FFHA in the order of their appearance in the primary sequence. While FocA, FdhC, NirC-β, NirC-γ and HSC subfamilies have FFHA in the central constriction, there is a slight variation in NirC-α with FFHV forming the central constriction. The uncharacterized YfdC subfamilies deviate from the above pattern. The residues YIHV form the central constriction in YfdC-α. YfdC-β channels display diversity with residues FLHL, FHLV and YIHV as part of the central constriction. We calculated the CHI and CVV values for all the prokaryotic FNT members. The CHI value of central constriction region for a typical FNT channel with residues FFHA is +4.2. Only members of NirC-α and some members of YfdC-β have central constriction slightly more hydrophobic with CHI values ranging from +6.6 to +7.6. As expected, distribution of CHI values calculated for all FNT channels shows a peak around +4.0 (data not shown). However, there are some examples indicating that some of the FNT channels are likely to have less hydrophobic and slightly more hydrophilic central constriction. We have found at least one example of an FNT channel which does not belong to any of the phylogenetically classified subgroups of FNT channel showing a CHI value of −2.8 (Fig. 6C) and the residues YFQS constitute the central constriction (Table 7). Several members of YfdC-α subfamily also have central constriction with CHI values negative ranging from −1.4 to −0.8 and these channels typically have Tyr and His residues along with Ser or Thr. At the other end of the spectrum, we have couple of examples from YfdC-β and from an unclassified FNT channel in which three out of four residues in the central constriction are bulky hydrophobic residues with CHI values +8.3 (Table 7). This analysis indicates that these FNT channels with unusual central constriction region with either more hydrophilic or hydrophobic regions are likely to facilitate the transport of solutes that will have chemical characteristics distinct from the solutes that are transported by the majority of FNT channels.

**Table 7:** FNT channels with unique central constriction region

FNT subfamily	UniProt ID^a^ (Organism)	Central constriction residues^b^	CHI/CVV value^c^
Unclassified	Q1Q4Z9 (*Candidatus Kuenenia stuttgartiensis*)	YFQS	CHI: −2.8
YfdC-α	Q0G7B8 *(Fulvimarina pelagi*)	YLHT	CHI: −1.4
YfdC-α	Q1YI18 (*Aurantimonas manganoxydans*)	YVHT	CHI: −1.0
YfdC-α	I1ARB3 (*Citreicella sp.*)	YMHA	CHI: −0.8
YfdC-β	K9AG69 (*Brevibacterium casei*)	FVHI	CHI: +8.3
Unclassified	Q1PZF5 (*Candidatus Kuenenia stuttgartiensis*)	FIHV	CHI: +8.3
NirC-γ	B9DL87, B9DLT5 (*Staphylococcus carnosus*) B9E993 (*Macrococcus caseolyticus*) E8SEZ7 (*Staphylococcus pseudintermedius*) E8SFH6 (*Staphylococcus pseudintermedius*) G5JLA8 (*Staphylococcus simiae*) I7JE48 (*Staphylococcus equorum*) K9AHL1, K9ANW4 (*Staphylococcus massiliensis*) J9HA25 (*Staphylococcus sp.*) and 6 more	FFHY	CVV: 529 Ǻ^3^

^a^The unique UniProt Accession ID is given

^b^The four residues that form central constriction site within the channel. They correspond to F75 from TM2a, F202 from TM5a, H209 from S-loop and A212 from TM5b of the formate channel (PDB ID: 3KCU)

^c^Calculation of Cumulative Hydropathy Index (CHI) and Cumulative vdw Volume (CVV) are described in the text

When CVV values were analyzed, we have found several examples of NirC-γ members in which all four residues of the central constriction are bulky aromatic residues and deviate significantly from the average value of 463.9 (±18.0) Ǻ^3^. Almost all these channels belong to the genus *Staphylococous* and many organisms from this genus are implicated in diseases like skin infection. A typical FNT channel has FFHA residues in the central constriction with CVV value 455 Ǻ^3^. However, these identified NirC-γ members have FFHY residues in the central constriction with the CHI value 529 Ǻ^3^ (Fig. 6F). The size restriction will force these channels to restrict the transport of larger solutes through these channels.

## dbFNT – A database for FNT family

**Fig. 7:**
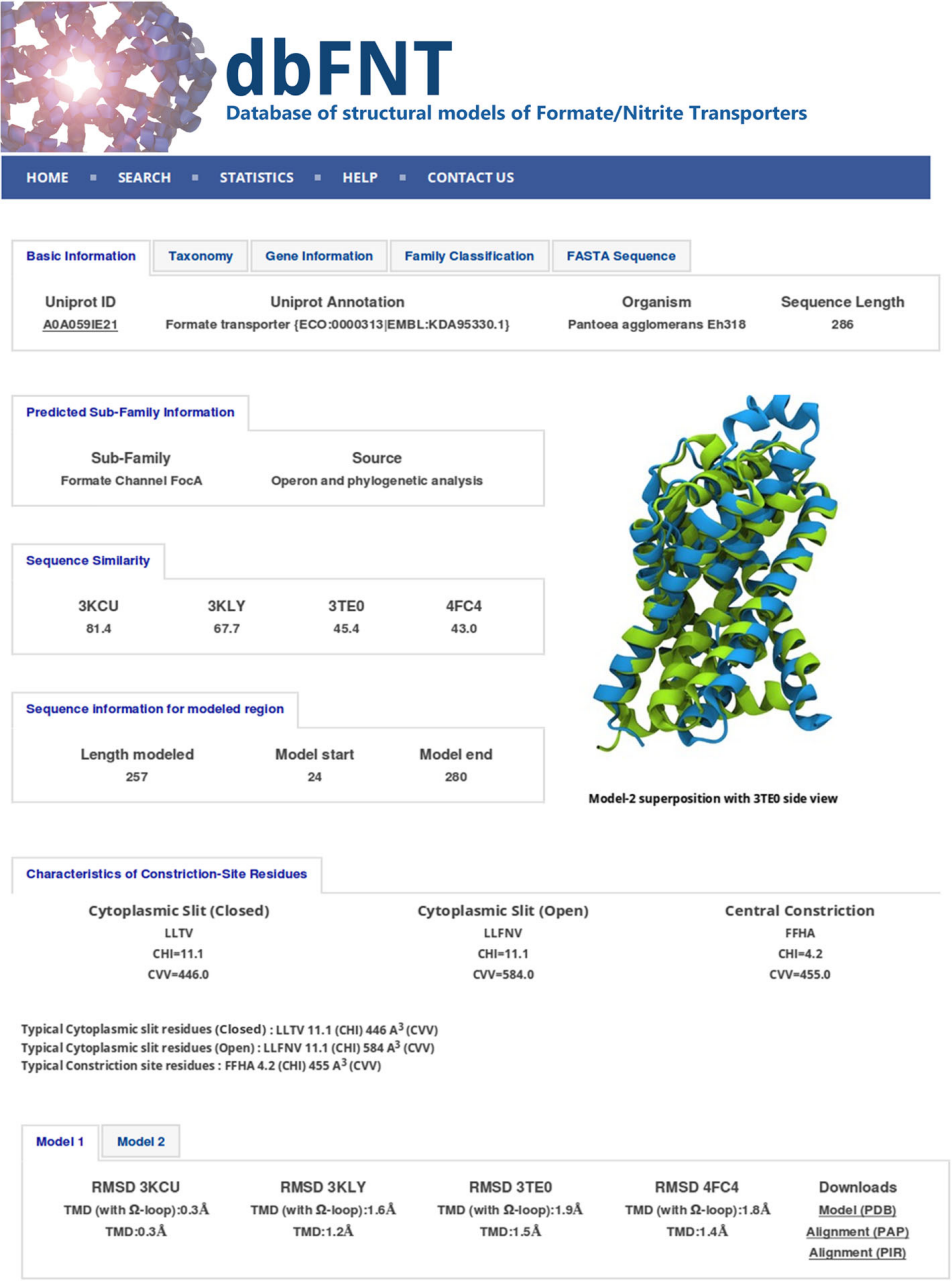
A sample record from dbFNT database for a member of FNT channel family. Screenshot showing the sample record for a FNT channel. In addition to details about FNT subfamily, its source and operon information (if available), the residues forming the two constriction regions along with their CHI and CVV values are also provided. Details about the multiple templates and the RMSD (in Ǻ) values of the model with respect to each template are available. The user can download the sequence and the modeled structures in FASTA and PDB formats respectively

We have constructed dbFNT, a database of FNT family which is freely available (see Additional file 4). MySQL5 and Python 2.6 have been used to manage and access the database. The user interface is designed using HTML, javascript and CSS scripts. The database currently contains information about more than 2200 FNT members from bacteria, archaea and eukaryotes. A sample entry from the database for one of the FNT sequences is shown in Fig. 7. Each FNT sequence has been assigned a unique ID called dbFNT ID. For each FNT member, this database provides details about the sequence, structural models, the FNT subfamily to which it belongs, the taxonomical details, gene ID, operon information (if available) and PFam, Prosite or InterPro classification. The structural models in PDB format can also be downloaded for further analysis. For the selected set of FNT sequences, the user can obtain structure-based sequence alignment with functionally and structurally important residues highlighted in color (see Additional File 5). There are also several search options available in the database. The user can search using the UniProt ID. One can also search using partial or full sequence of a known FNT member. The “Advanced Search” options include search based on residues of the two constriction regions or organism (see Additional File 6). One can also restrict the search only to the FNT sequences belonging to a particular subfamily. The dbFNT database is freely available to the scientific community at http://bioinfo.iitk.ac.in/dbFNT.

## Conclusions

Members of the formate/nitrate transporters (FNTs) family that are selective for monovalent anions share the hour-glass helical fold with the major intrinsic protein (MIP) family which transports neutral solutes. Some of the MIP family members from bacteria, plants and mammals have been extensively studied and are well characterized using mutation studies, structural and computational techniques [31, 44–50]. In comparison with MIP members, FNT channels have not been characterized well and the mechanism of selectivity/transport of anions and the role of different regions of the channel are yet to be clearly ascertained. The absence of FNTs in humans should have made these proteins as prime candidates for drug targets and it is surprising that the FNT family has not attracted enough attention in this direction. With many examples of FNT members present in several human pathogens, it is hoped that more focus will be given to the understanding of FNT channel’s structure-function relationship. With this aim, the present study has compiled and analyzed more than 2000 FNT sequences from diverse organisms. In the first analysis, we examined the conservation of residues by including all the FNT sequences including those from eukaryotes. We found that one third of all transmembrane positions show conservation and the residues are from the constriction sites or channel-facing or at the helix-helix interface. We then considered only the prokaryotic FNT sequences. The phylogenetic analyses helped in the identification of eight different FNT subfamilies in prokaryotes. The classification of clusters was supported by operon-based analysis and taxonomic distribution specific to the subfamilies. Further, structure-based sequence alignments helped to recognize subfamily-specific residue and group conservation. We have also identified specific FNT members that have unique central constriction or cytoplasmic slit. Availability of these details along with the structural models in the dbFNT database will facilitate the experimental researchers to design mutational studies that can help in elucidating the mechanism of an individual FNT channel’s selectivity and transport of anions. The study of substrates of FNT members with unusual features, the role of residues showing subfamily-specific conservation and the gating mechanism of these channels require an interdisciplinary approach combining the experimental and computational expertise.

## Methods

### Identification of FNT channel members

The UniProtKB (www.uniprot.org) database [33] was initially queried for Formate/Nitrite Transporter (FNT) proteins from all organisms. Family and domain database cross-references were used for this purpose. We looked for PROSITE [51] entries PS01005 and PS01006, PFam [52] accession ID PF01226 and InterPro [53] entries IPR000292, IPR024002 and IPR023999 in the cross-references. We only considered those sequences with at least 150 residues long since each FNT channel will have six transmembrane segments. The redundancy of identified sequences from UniProt was removed at 98% identity cut-off using the program CD-HIT [54, 55]. However, sequences from different species with more than 98% sequence identity were retained. The sequences thus extracted were further checked for the presence of six transmembrane segments. TMHMM [56], Phobius [57] and HMMTOP [58] were used to examine whether each sequence has at least six putative transmembrane segments. Presence of FNT family signatures and conservation of selectivity filter residues were verified by generating multiple sequence alignment using the program PRALINE [59] . The alignments were guided by four FNT members for which three-dimensional structures have been determined (PDB IDs: 3KCU [25], 3KLY [26], 3TDR [11] and 4FC4 [28]). Any sequence lacking the requisite number of transmembrane segments and/or features characteristic of FNT family as described above were discarded.

### Operon prediction for FNT members

To increase the confidence level for the identified putative FNT members from sequence search, we exploited the fact that FNT members are genetically linked to their partner enzymes and they occur together as part of the same operon. For example, formate channels are linked with pyruvate formate lyase (*pflBA*) or formate dehydrogenase (*fdhAB*) [8, 60, 61]. Nitrite and hydrogen sulfide channels are encoded along with nitrite reductase and sulfite reductase forming part of the operons *nirBD* [1, 62] and *asrABC* [11, 63] respectively. Thus for an FNT member, if details are available at genomic level, then that information was used to validate the functional annotation and subfamily classification. The following procedure was adopted for this purpose.

Genomic annotation was extracted in the form of feature tables from NCBI Genome database (http://www.ncbi.nlm.nih.gov/genome). For each FNT member, the gene and transcript annotations of at least three neighboring genes both upstream and downstream of the same transcriptional strand were searched for the respective partner enzymes. If there is no annotation for the gene transcript, then the transcribed protein was used as a query in BLAST [64] to search the local database of sequentially diverse representatives of known operon members. We used a consistent definition for the operon as described in previous literature.

### Homology modeling

The three-dimensional structure of each FNT member was constructed using MODELLER v9.13 [65, 66]. The modeling protocol is similar to the one developed earlier in our laboratory to model the members of major intrinsic proteins (MIPs) [34–36]. However unlike in the earlier approach in which the same protocol was used to model all MIP members, here we have adopted a slightly different approach due to certain structural differences observed in specific regions such as the functionally important Ω-loop and the N-terminal region of some of the FNT members. We used a strategy which is subfamily-specific so that the two different open and closed conformations could be modeled for members belonging to FocA clade. The following crystal structures were used as templates. Structures of formate channel FocA from *E. coli* (*EcFocA*; PDB ID: 3KCU [25], Chain B) and *V. cholerae* (*VcFocA*; PDB ID: 3KLY [26], Chain E), hydrogen sulphide channel HSC from *Peptoclostridium difficile* (*PdHSC*; PDB ID: 3TE0 [11], Chain E) and nitrite channel NirC from *Salmonella enterica* (*SeNirC*; PDB ID: 4FC4 [28], Chain I) were used as templates. Since FNT channels form pentamers, only one monomer from the experimentally determined structures was used to model FNT sequences. The generated models correspond to physiological pH. Structures of *EcFocA* and *VcFocA* were used to model FocA family members in open and closed conformations respectively. Since N-terminal region is disordered at physiological pH in both the template structures, modeling of N-terminal region was not included for FocA members. Regions of the N-terminal helix and the Ω-loop in fully helical form in the hydrogen sulphide channels were modeled using *PdHSC* and *SeNirC* channel structures as templates. For all other FNT members, all four template structures were used to model the Ω-loop region and without the N-terminal fragment.

Extreme care was taken to avoid gaps in the transmembrane regions by manually examining the target-template alignment. Highly conserved residues and motifs as identified in databases like PROSITE [51] were also used to guide proper alignments. We built a total of ten models for each FNT member and the best model with optimum MODELLER objective function was selected for further refinement. MODELLER’s loop optimization protocol and the graph theory-based SCWRL4 [67] were used to refine the loops and side-chain conformations respectively. The resultant model was energy minimized using GROMACS v5.0.6 [68] with the CHARMM36 force-field. Finally, PROCHECK [69] was used to check the quality of each FNT model.

### Phylogenetic analysis

We performed a detailed phylogenetic analysis on all the extracted FNT members to identify the different sub-families and to understand the diverse nature FNT family members. We used two different clustering methods, namely, maximum likelihood (ML) using RAxML v8.1 (HPC_MPI version) [37] and Bayesian inference using MrBayes v.3.2.4 [38]. The structure-based alignment obtained for the transmembrane helical segments obtained from the homology models were used as inputs for both methods. More than 2000 prokaryotic FNT members were analyzed.

Among the 20 available substitution models in RAxML, the LGF model provided the best log likelihood score for the input multiple sequence alignment (MSA). The rate heterogeneity across sites was approximated with the gamma-distribution. The reliability of individual branches of the best ML tree was determined using 1000 rapid bootstrap replicates. The final trees were analyzed at 50% confidence level. Various sub-family groupings were then derived based on the resultant trees with the above mentioned criteria.

To validate different groupings found in ML method, we independently performed Bayesian analysis using MrBayes and the WAG model proposed by Whelan and Goldman [70] was applied. Two separate runs were performed for the input MSA. Convergence was tested using three different parameters, namely, average standard deviation of split frequency (ASDSF), potential scale reduction factor (PSRF) and the average estimated sampling size (ESS). The conditions used for convergence are as follows: ASDSF reached less than 0.1 for the prokaryotic dataset [38]; PSRF was nearly 1 for all parameters [71]; ESS was at least 100. To reach convergence, the prokaryotic datasets required at least 6 million generations. The number of prokaryotic FNT sequences resolved poorly is 320 and hence they were discarded. A reduced tree for 1723 prokaryotic FNT sequences was recalculated and validated using both methods. This tree was used for final sub-family groupings and further sub-family-based analyses were performed. Wherever it is possible, operon information and known features specific to a sub-family were used to classify and confirm the phylogenetic groupings.

## Additional files

Additional file 1: Figure S1. (A) Positions of small and weakly polar residues showing very high group conservation. (B) Residues facing the channel interior that exhibit high level of conservation across all 2206 FNT channels. (DOC 1156 kb)

Additional file 2: Table S1. Positions showing high conservation of hydrophobic or aromatic character of residues that are either lipid-exposed or at the monomer-monomer interface. (DOC 46 kb)

Additional file 3: Table S2. Average lengths of loops connecting the transmembrane segments and termini regions for different FNT subgroups. (DOC 29 kb)

Additional file 4: Screenshot of dbFNT homepage page. (PNG 1345 kb)

Additional file 5: Screenshot of dbFNT webpage showing the structure-based sequence alignment for selected FNT channels. (PNG 2147 kb)

Additional file 6: Screenshot of dbFNT search page showing different search options. (PNG 638 kb)

## Abbreviations

ASDSF: Average standard deviation of split frequency
CHI: Cumulative Hydropathy Index
CVV: Cumulative van der Waals Volume
ESS: Estimated sample size
FNTs: Formate/Nitrate Transporters;
ML: Maximum likelihood
MSA: Multiple sequence alignment
PDB: Protein Data Bank
PSRF: Potential scale reduction factor
