# Supplementary material for: Anion-selective Formate/nitrite transporters: taxonomic distribution, phylogenetic analysis and subfamily-specific conservation pattern in prokaryotes
Source: BMC Genomics. 2017 Jul 24;18:560. doi: 10.1186/s12864-017-3947-4 (PMC5525234; doi:10.1186/s12864-017-3947-4)
Supplement: Supplementary file 1 — (A) Positions of small and weakly polar residues showing very high group conservation. (B) Residues facing the channel interior that exhibit high level of conservation across all 2206 FNT channels. (DOC 1156 kb) [file 12864_2017_3947_MOESM1_ESM.doc]

**Figure S1**


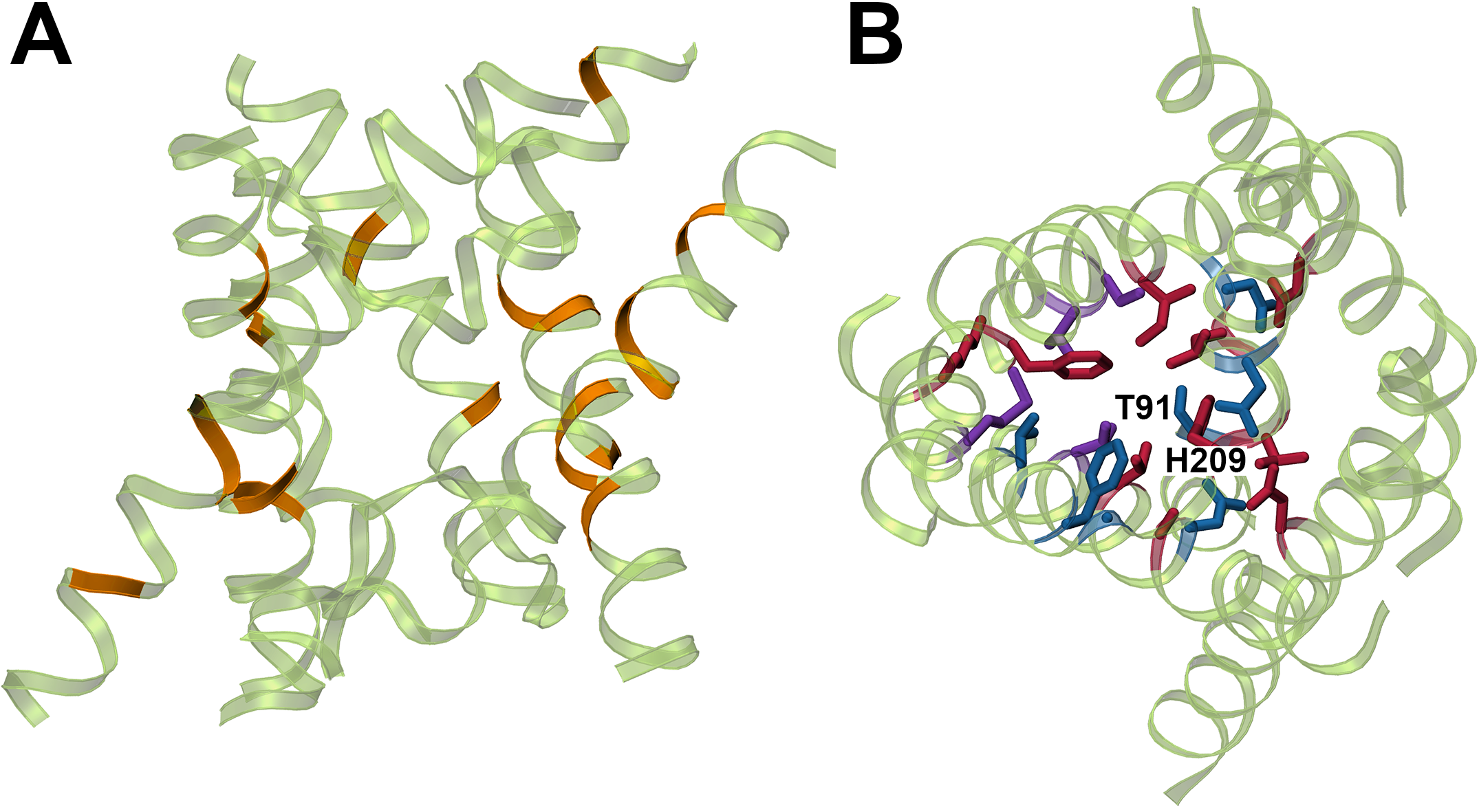


**Figure S1: Conservation across all FNT channels from bacteria, archaea and eukaryotes**

(A) Positions of small and weakly polar residues showing very high group conservation are shown in brown. (B) Residues facing the channel interior that exhibit high level of conservation across all 2206 FNT channels. Residues are colored according to the percentage conservation (red: >95%; purple: 85-95%; blue: 75-85%). Details of residue positions showing high group conservation of small and weakly polar residues and conservation of channel facing residues can be found in Tables 1 and 2 respectively.
