# Supplementary material for: Anion-selective Formate/nitrite transporters: taxonomic distribution, phylogenetic analysis and subfamily-specific conservation pattern in prokaryotes
Source: BMC Genomics. 2017 Jul 24;18:560. doi: 10.1186/s12864-017-3947-4 (PMC5525234; doi:10.1186/s12864-017-3947-4)
Supplement: Supplementary file 2 — Positions showing high conservation of hydrophobic or aromatic character of residues that are either lipid-exposed or at the monomer-monomer interface. (DOC 46 kb) [file 12864_2017_3947_MOESM2_ESM.doc]

**Table S1**: Positions showing high conservation of hydrophobic or aromatic character in FNT channels^a^

| Residue^b^ | Location within the channel | Residues in the position^c^ | Conservation (%)^d^ | Remarks |
| --- | --- | --- | --- | --- |
| L69 (TM2a) | Facing the mouth of the central pore | L(53), V, M, I | 78.4 | Hydrophobic |
| L77 (TM2a) | Monomer-monomer interface | L, I, V, M | 75.8 | Hydrophobic |
| I80 (TM2a) | Monomer-monomer interface | I(33), V, M, L | 76.2 | Hydrophobic |
| L81 (TM2a) | Monomer-monomer interface | L(46), M, I, V | 77.6 | Hydrophobic |
| V84 (TM2a) | Monomer-monomer interface | L(37), I, M, V | 82.4 | Hydrophobic |
| L95 (Ω-loop) | Monomer-monomer interface | L(39), M(33), V, I | 83.8 | Hydrophobic |
| W114 (TM3) | Membrane-water interface | W(77), F | 79.4 | Aromatic |
| V123 (TM3) | Lipid-exposed | I(33), L, V | 76.1 | Hydrophobic |
| L126 (TM3) | Lipid-exposed | L(42), I, V, M | 75.2 | Hydrophobic |
| I169 (TM4) | Lipid-exposed | I(64), V, L, M | 93.8 | Hydrophobic |
| M174 (TM4) | Monomer-monomer interface | L(52), M, I, V | 87.8 | Hydrophobic |
| L177 (TM4) | Monomer-monomer interface | L(56), V, I, M | 75.8 | Hydrophobic |
| D190 (TM5a) | Facing the mouth of central pore | G(39), A, S, T | 81.7 | Small and weakly polar residues |
| A192 (TM5a) | Monomer-monomer interface | I(35), V, L, M | 77.0 | Hydrophobic |
| F207 (S-loop) | Monomer-monomer interface | F(65), Y | 78.7 | Hydrophobic |
| I211 (TM5b) | Away from the channel | V(60), I(37) | 97.5 | Hydrophobic |
| I221 (TM5b) | Lipid-facing | I(34), V, L, M | 81.7 | Hydrophobic |
| I256 (TM6) | Lipid-exposed | I(44), L, V | 83.4 | Hydrophobic |
| I260 (TM6) | Lipid-exposed | L(53), I, V | 84.7 | Hydrophobic |
| I263 (TM6) | Lipid-exposed | I(50), V, L, M | 76.6 | Hydrophobic |
| I264 (TM6) | Lipid-exposed | I(42), V(39), L | 90.9 | Hydrophobic |

^a^All 2206 FNT channels from bacteria, archaea and eukaryotes were considered for this analysis

^b^Residue numbers correspond to that of formate channel structure as available in the PDB ID: 3KCU. Occurrence of these residues in the transmembrane segments or the functionally important loop region is also provided

^c^Percentage conservation of the most frequently observed residue(s) is indicated in brackets

^d^Percentage conservation as observed in all 2206 FNT channels
