# Supplementary material for: Anion-selective Formate/nitrite transporters: taxonomic distribution, phylogenetic analysis and subfamily-specific conservation pattern in prokaryotes
Source: BMC Genomics. 2017 Jul 24;18:560. doi: 10.1186/s12864-017-3947-4 (PMC5525234; doi:10.1186/s12864-017-3947-4)
Supplement: Supplementary file 3 — Average lengths of loops connecting the transmembrane segments and termini regions for different FNT subgroups. (DOC 29 kb) [file 12864_2017_3947_MOESM3_ESM.doc]

**Table S2**: Average lengths of loops connecting the transmembrane segments and termini regions for different FNT subgroups^a^

| Region^b^ | FocA | FdhC | NirC-α | NirC-β | NirC-γ | HSC | YfdC-α | YfdC-β |
| --- | --- | --- | --- | --- | --- | --- | --- | --- |
| N-ter | 32 (14) | 26 (7) | 25 (8) | 25 (9) | 29 (9) | 25 (7) | **55 (23)** | **42 (20)** |
| Loop -A | 7 (1) | 7 (2) | **1 (1)** | 4 (0) | 8 (2) | 7 (1) | 5 (2) | 6 (3) |
| Loop-B | 5 (0) | 6 (0) | 8 (1) | 6 (0) | 6 (0) | 6 (0) | 7 (1) | 6 (1) |
| Loop-C | 26 (1) | 27 (6) | **35 (1)** | 24 (1) | 24 (1) | 24 (1) | 23 (0) | 23 (1) |
| Loop-D | 3 (0) | 3 (0) | 3 (0) | 2 (0) | 3 (1) | 3 (0) | 1 (1) | 3 (0) |
| Loop-E | **22 (1)** | 3 (3) | 4 (0) | 5 (0) | 6 (2) | 5 (2) | 3 (1) | 3 (0) |
| C-Ter | **47 (78)** | 17 (9) | 20 (8) | 23 (2) | 12 (3) | 16 (11) | **69 (116)** | 15 (7) |

^a^Average lengths of loop and termini regions along with standard deviation (in brackets) are given for each FNT subfamily. The figures shown in bold belong to the subfamilies that show significant differences in the lengths from other subfamilies

^b^N-ter: Segment before TM1; Loop A, Loop-B, Loop-C, Loop-D and Loop-E respectively represent the regions connecting TM1-TM2, TM2-TM3, TM3-TM4, TM4-TM5 and TM5-TM6; C-ter: Segment after TM6
