# Supplementary figures and images for: Anion-selective Formate/nitrite transporters: taxonomic distribution, phylogenetic analysis and subfamily-specific conservation pattern in prokaryotes
Source: BMC Genomics. 2017 Jul 24;18:560. doi: 10.1186/s12864-017-3947-4 (PMC5525234; doi:10.1186/s12864-017-3947-4)

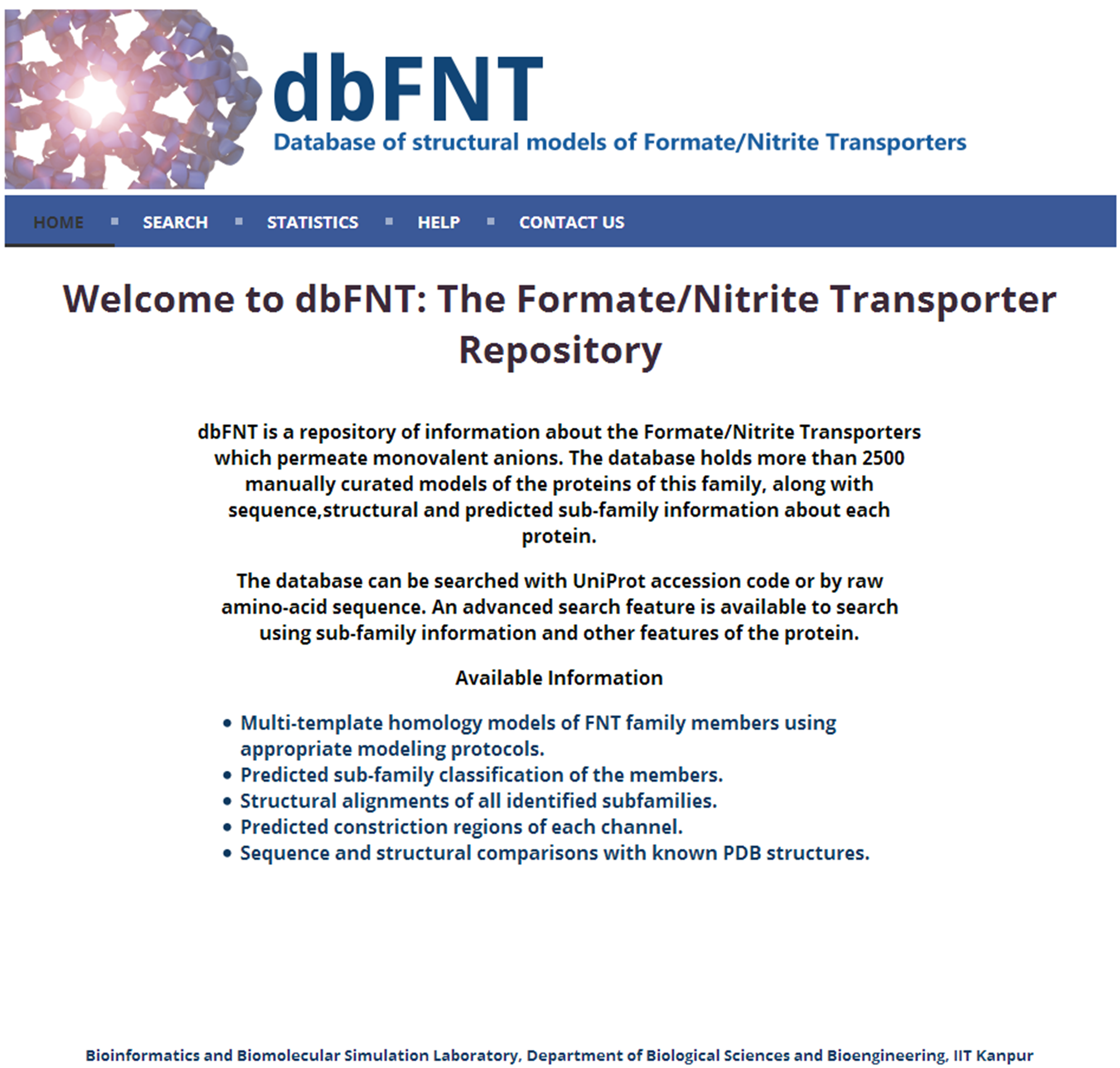

Supplement: Supplementary file 4 — Screenshot of dbFNT homepage page. (PNG 1345 kb) [file 12864_2017_3947_MOESM4_ESM.png]

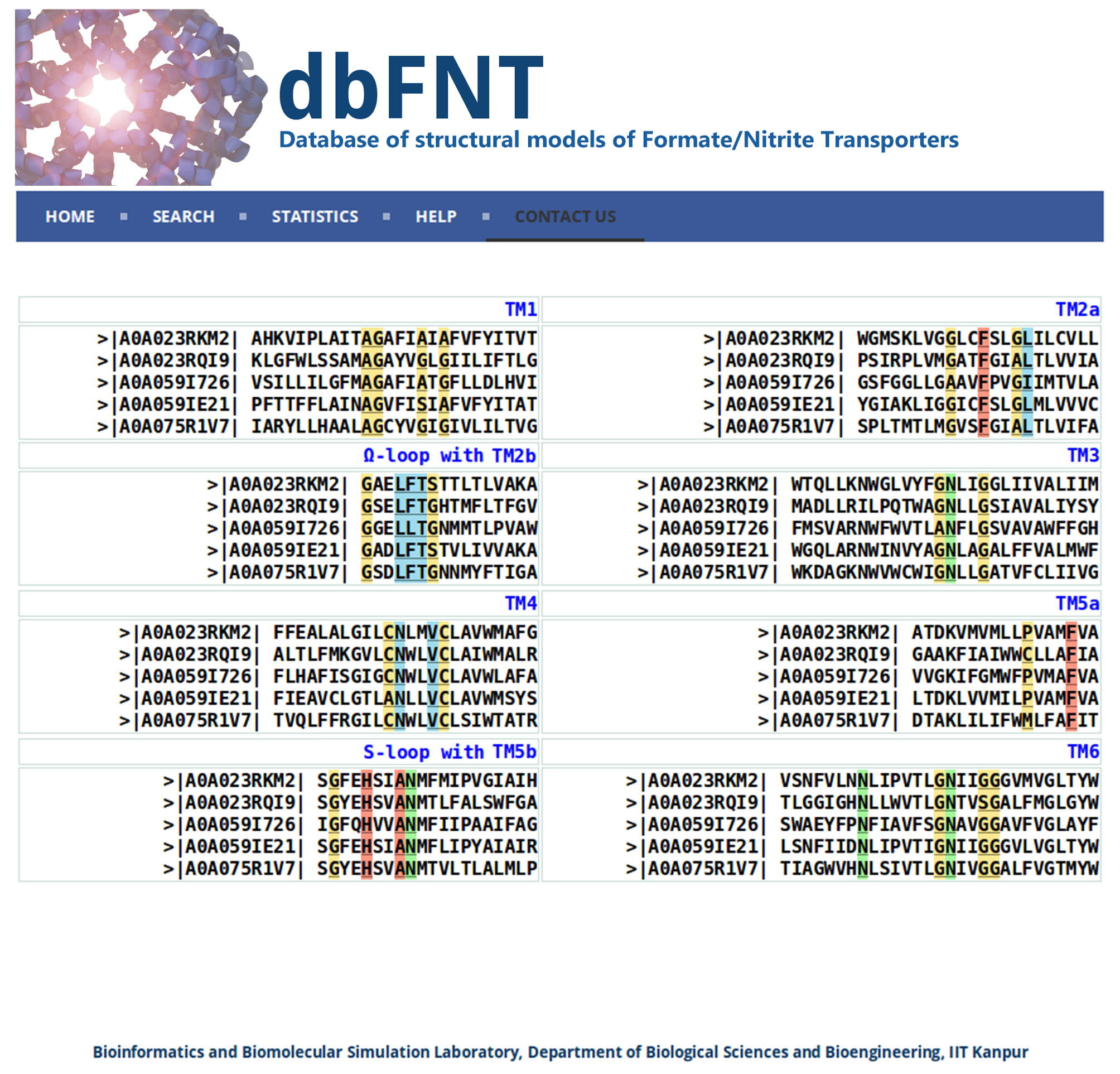

Supplement: Supplementary file 5 — Screenshot of dbFNT webpage showing the structure-based sequence alignment for selected FNT channels. (PNG 2147 kb) [file 12864_2017_3947_MOESM5_ESM.png]

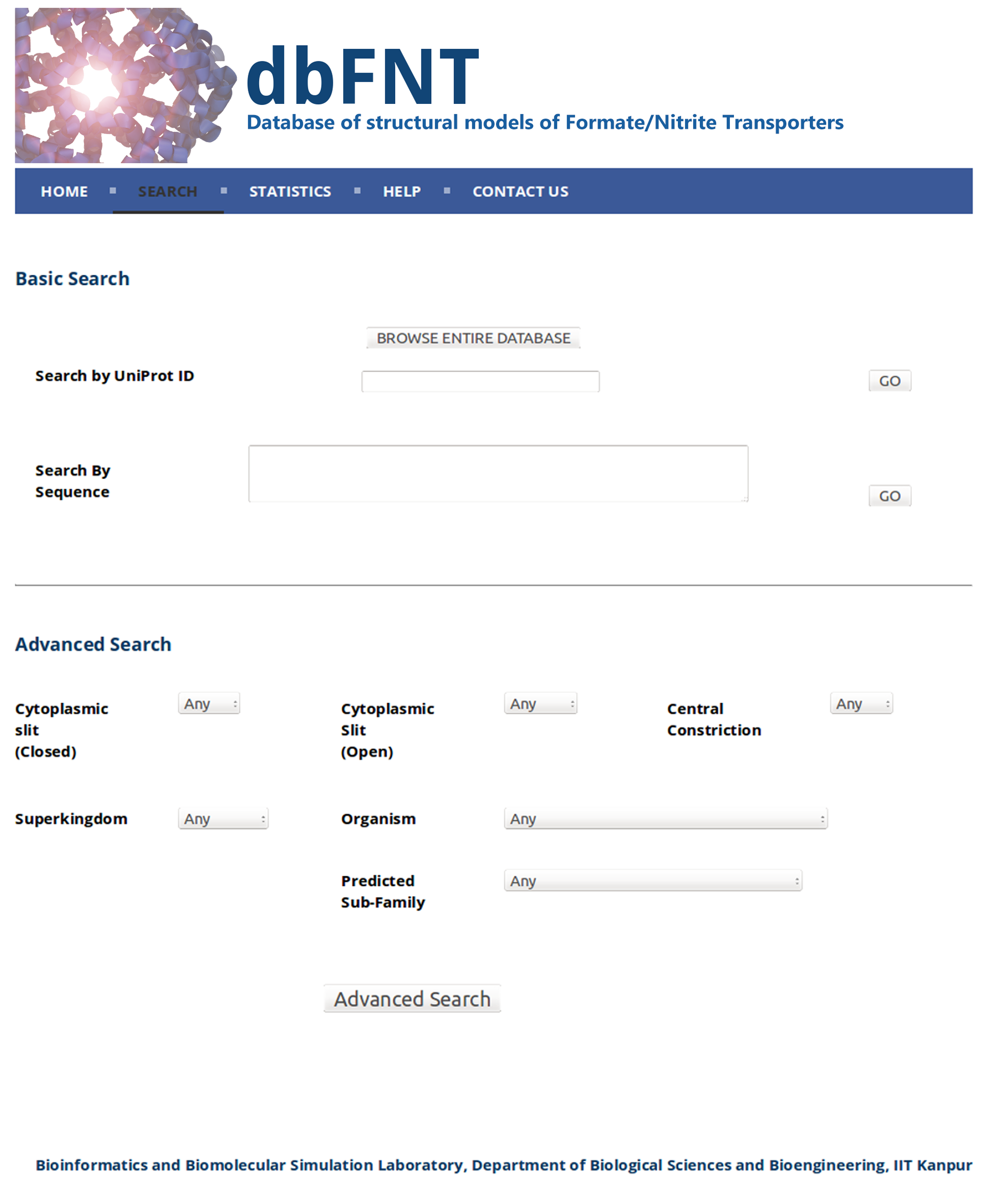

Supplement: Supplementary file 6 — Screenshot of dbFNT search page showing different search options. (PNG 638 kb) [file 12864_2017_3947_MOESM6_ESM.png]
